# Changing Flight and Flocking Dynamics of Homing Pigeons (*Columba livia d*.) Over Heterogeneous Landscapes

**DOI:** 10.1002/ece3.71902

**Published:** 2025-08-06

**Authors:** Robin S. Mehlhausen‐Franks, Steven J. Portugal

**Affiliations:** ^1^ School of Biological Sciences Royal Holloway University of London Egham UK; ^2^ Department of Biology University of Oxford Oxford UK

**Keywords:** adaptation, behaviour, flap, frequency, habitat

## Abstract

With the global rapid expansion of urban and developed areas, an understanding of how species adapt behaviourally and physiologically to changing environments is of ever‐increasing importance. Anthropogenic land development is of particular significance to species that traverse long distances in groups, such as migratory birds. Despite the high energetic cost of powered flight, there has been little research into how bird species adapt their flight patterns in response to changes in topography. Notably, there remains a gap in our understanding of how terrain cover impacts the energetic cost of flight. We examined several parameters describing flight and flock behaviours in three cluster flocks of homing pigeons (*Columba livia d*.), including flap frequency as a proxy for energy expenditure. Each flock was flown repeatedly over a heterogeneous landscape of open, wooded, and urban habitats. We found that the birds adopted significantly slower flight and less dense flocking behaviour when traversing over woodland, while flying significantly faster with a lower flap frequency over urban areas. The causes of these trends are not immediately clear, and we discuss a range of potential explanations, including the influence habitat has on the wariness of individuals and the significance of landmarks and visual noise in aerial navigation.

## Graphical abstract

Using GPS and accelerometry biologgers, we investigated how the flight and group behaviour of flocking homing pigeons changed in response to flying over a patchwork landscape of open, wooded and urban terrain. Key parameters included flap frequency, flight speed and flock density measures. We found that flocking birds flew significantly slower and flocked significantly less densely over woodland, while they flew significantly faster with a lower flap frequency over urban areas.

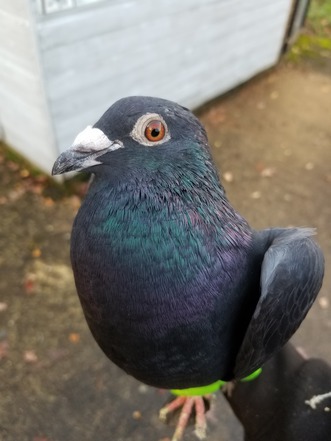

## 1 Introduction

Among the most widespread behaviours in nature is the congregation of individuals into groups, driven by environmental pressures and individual needs (Emlen 1952; Wilson 1980; Underwood 1982; Creel and Creel 1995; Grünbaum 1998; Krause and Ruxton 2002; Giardina 2008; Krause et al. 2009). Grouping can be found in some form in both single‐cell and multi‐cellular organisms (Buhl et al. 2006; Bajec and Heppner 2009; Ward and Webster 2016a), with the associated benefits and costs varying over species, context and the different needs, physiological conditions, and personalities of constituent individuals (Franks 1986; Krause and Godin 1995; Krause and Ruxton 2002; Usherwood et al. 2011; Aplin et al. 2014; Hansen et al. 2015; Olson et al. 2015; Ward and Webster 2016b; Hintz and Lonzarich 2018). The benefits of grouping usually represent ‘tangible’ incentives at the individual level through improved survivability against predation (Treherne and Foster 1982; Morgan and Godin 1985; Cresswell 1994; Blumstein 2013; Lehtonen and Jaatinen 2016), reducing the cost of searching for viable mates (Wiley 1978; Clutton‐Brock et al. 1988; Robbins 1999; Barnard 2000; Romey and Wallace 2007; Ward and Webster 2016b) and increasing the rate of energy uptake through foraging or hunting (Bautista et al. 1998; Barnard 2000; Beauchamp 2005; Aplin et al. 2014; Olson et al. 2015; Heldbjerg et al. 2017; Giraldeau and Caraco 2018). Interactions between individuals, and with their environment, often give rise to a broad array of unique behaviours and movement dynamics within animal groups (Weaver 1966; Giraldeau and Lefebvre 1986; Aubin et al. 2000; Conte and Hefetz 2008; Ord and Garcia‐Porta 2012; Ward and Webster 2016b; Kappeler 2019). Fischer et al. (2017) demonstrated that the behavioural development of individual cichlid fish was significantly affected by different social contexts and at different levels of perceived predator threat. Similarly, Bode et al. (2010) found that perceived threat significantly influenced three‐spined sticklebacks ( *Gasterosteus aculeatus* ) to adopt more synchronised group movements. Bode et al. (2010) attributed this change in behaviour to the oddity effect, wherein individuals who are easy for predators to distinguish from their groupmates are subject to an increased attack rate (Landeau and Terborgh 1986; Krause and Ruxton 2002).

External pressures that drive the way in which groups move through space are among the most important to understand, as any changes in the environment through which animals traverse can have significant ramifications for their fitness (Viscido and Wethey 2002; Radford 2004; Dumont et al. 2005; Fahrig 2007; Klaassen et al. 2011; Bode et al. 2012; Della‐Rossa et al. 2013; Nakayama et al. 2016; Taylor et al. 2020). The energetics of movement and navigation are potentially more important to bird flocks than any other form of animal group, given the high cost of powered flight (Tucker and Catlett 1973; Tucker 1975; Alexander 2003). Bird flocks can be roughly categorised into two types: linear, principally the V‐shaped flocks of species such as pink‐footed geese (*Anser brachyrhynchis*; Cutts and Speakman 1994; Klaassen et al. 2006) and white storks ( *Ciconia ciconia* ; Flack et al. 2018) and cluster or globular flocks, such as in the murmurations of common starlings ( *Sturnus vulgaris vulgaris* ; Carere et al. 2009; Vīgants et al. 2023). In contrast to the shared benefits of improved navigational efficiency and predator defense (Simons 2003; Dell'Ariccia et al. 2008; Freeman and Biro 2009; Flack 2013; Flack, Freeman, et al. 2013; Mueller et al. 2013; Berdahl et al. 2018; Newton 2024), the two categories of flocks confer vastly different aerodynamic environments. V‐formation flocks reduce the cost of flight for individuals by conferring reduced drag and increased lift (Badgerow and Hainsworth 1981; Cutts and Speakman 1994; Portugal et al. 2014; Voelkl et al. 2015; Mirzaeinia et al. 2020; Portugal 2020), whereas conversely cluster‐flocking necessitates an increase in flap frequency and thus energetic cost. Close proximity to multiple neighbours in a constantly moving group requires a high degree of stability for maintaining synchronicity with neighbours and to avoid collisions, which results in higher energy expenditure (Sansom et al. 2008; Usherwood et al. 2011; Sankey and Portugal 2019; Taylor et al. 2019; Portugal 2020).

Regardless of flock type, bird groups have been observed to significantly alter their flight paths in response to various topologies, typically associated with different benefits or dangers (Bélisle et al. 2001; Rodríguez et al. 2001; Whittingham and Evans 2004; Carrascal and Alonso 2006; Fahrig 2007; Diehl 2013). For example, white storks are known to be reliant on thermal updrafts rising from open spaces to facilitate their migratory flights (Scacco et al. 2019), and through this mechanism many soaring bird species increase their flight ranges from 22% to 91% (Leshem and Yom‐Tov 1996). Conversely, passerine species of the *Parus* genus have been shown to prefer denser and more mature boreal forest over open space due to the woodland offering superior cover from predators (Rodríguez et al. 2001). Garde et al. (2021) showed that flocks of homing pigeons (*Columba livia domestica*) alter their flight paths in anticipation of changing topography, minimising the time spent ascending in order to reduce energetic expenditure during flight. While Garde et al.'s (2021) work was pioneering in linking the flight behaviour of individual birds and flocks to ground topology and vegetative cover at a high resolution, they do not provide a direct measure of individual flight effort or the associated energetic costs.

The cost of powered flight can be effectively quantified using flap, or wingbeat, frequency (Pennycuick 1990, 1996; Zhang 2017). Changes in the frequency of wingbeat oscillations made by an individual can be tracked over the course of individual flights at high spatial resolutions using accelerometery biologgers and measured as ‘wingbeat‐’ or ‘flap frequency’ (Pennycuick 1996; Schmaljohann and Liechti 2009; Sankey and Portugal 2019; Taylor et al. 2019). Flap frequency offers a high‐resolution and reliable tracer of individual flight effort unlike, for example, flight speed, whose relationship with the cost of flight is inconsistent and varies between contexts (Pennycuick 1996; Ling et al. 2018; Sankey and Portugal 2019; Sankey et al. 2019; Taylor et al. 2019). Given its variability, an understanding of how flight speed interacts with the cost of flight, within the changing environmental context of long‐distance flight, would offer valuable insight into the individual‐level processes driving flock and individual movement. Domesticated homing pigeons, as used by Garde et al. (2021), provide an ideal model species for studies into flight that combine behavioural, environmental, and physiological factors (McGregor and Haselgrove 2010). As a species that forms globular cluster flocks at both large and small scales (Tamm 1980; Taylor et al. 2019; Kano et al. 2021; Sankey et al. 2021), research into homing pigeon behaviour and physiology offers insight into the navigation (Freeman and Biro 2009; Flack 2013; Flack, Freeman, et al. 2013), flight and flocking behaviour (Pearson 1964; de Perera and Guilford 1999; Pettit et al. 2015; Sankey and Portugal 2019, 2023; Garde et al. 2021), and role of individual personality in animal groups (Biro et al. 2006; Flack et al. 2012; Jorge and Marques 2012; Flack, Ákos, et al. 2013; Santos et al. 2014, 2015; Pettit et al. 2015; Chen et al. 2016; Portugal et al. 2017; Sasaki et al. 2018; Gao et al. 2021).

Studies into cluster flocking utilise a range of variables to calculate descriptors of the condition, geometry, and behaviour of flocks; a key example being measures of flock density (Fernández‐Juricic et al. 2004; Usherwood et al. 2011; Sankey and Portugal 2019). Flock density has been shown to change in response to perceived predation risk (Ballerini et al. 2008; Aplin et al. 2014; Hogan et al. 2017), and over the course of time within flights (Sankey and Portugal 2019). Density has also been shown to correlate with flap frequency, and thus flight effort (Usherwood et al. 2011; Taylor et al. 2019). Another key parameter is the position of individuals within the flock. Positioning is relevant not only for protection from predators (Viscido and Wethey 2002; De Vos and O'Riain 2010; Aplin et al. 2014) but may also affect the aerodynamic environment formed in globular flocks, suggesting there may be preference for certain energetically beneficial positions within the flock (Usherwood et al. 2011; Portugal et al. 2014; Taylor et al. 2019; Portugal 2020). Flocking and flight parameters, including those described above, may also change significantly over short time spans during the course of an individual flight (Taylor et al. 2017, 2019; Sankey and Portugal 2019; Garde et al. 2021), or across multiple repeated flights as a result of increasing route familiarity (Meade et al. 2006; Pettit et al. 2013, 2015). Investigating the links (or lack thereof) between temporal and spatial changes is of particular relevance when studying flight over heterogeneous habitats, given that birds cross over multiple habitats in the short term and may be subject to changing land development in the long term.

With the increasing expansion of urban and developed areas (Maclean 2010; Soulsbury et al. 2015), many species struggle to adapt to rapidly changing environments (Batten 1972; Gaston 2010; Sol et al. 2017, 2020). Garde et al. (2021) analysed changes in a number of flight parameters over open ground and wooded space, which has been a common comparison in past research (Bélisle et al. 2001; Whittingham and Evans 2004; Carrascal and Alonso 2006). We set out to implement a third category for consideration: urban landscapes. Where woodland may provide havens for predators of homing pigeons (Götmark and Post 1997; Dixon 2002; Henderson et al. 2004), man‐made structures may provide preferable terrain due to their resemblance to ancestral cliff‐nesting breeding grounds (Lundholm and Richardson 2010; Ferretti 2019) and many species, including homing pigeons, have adapted to these environments (Bird et al. 1996; Clergeau et al. 1998; Parsons et al. 2006; Evans et al. 2011; Le Gros et al. 2016; Washburn et al. 2016; Teglhøj 2017).

Our overall aim was to determine if the flight and flocking behaviours of homing pigeon flocks change in response to open, wooded, or urban ground cover. We expected there to be a trend of slower, low‐powered flight over urban areas, contrasted by high‐cost, high‐speed, and high‐density flight over woodland, with open areas acting as a ‘middle‐ground’. Our interest was specifically in assessing changes in flap frequency, flock density, individual position within flocks, and flight speed over repeated flights through a heterogeneous landscape. Our core hypotheses were: (1) Flap frequency will significantly increase when flying over woodland and significantly reduce over urban areas, compared with open terrain, as a result of birds prioritising high‐agility defensive flight patterns in response to a perceived increased risk of being predated upon. (2) Flock density will significantly increase over woodland and significantly decrease over urban areas, compared with open terrain, as birds prioritise denser, more defensive flocking behaviour in response to a perceived increase in the risk of being predated upon. (3) Flight speed will significantly increase over woodland and significantly decrease over urban areas, compared with open terrain, as birds prioritise anti‐predator flight patterns and minimise time spent flying over areas of perceived increased predator risk. (4) Flock position relative to the direction of travel will not significantly change over different habitat ground cover categories, due to the cost of flight and flock leadership more strongly determining individual position than the threat of being predated upon.

## 2 Materials and Methods

### 2.1 Birds and Housing

**FIGURE 1:**
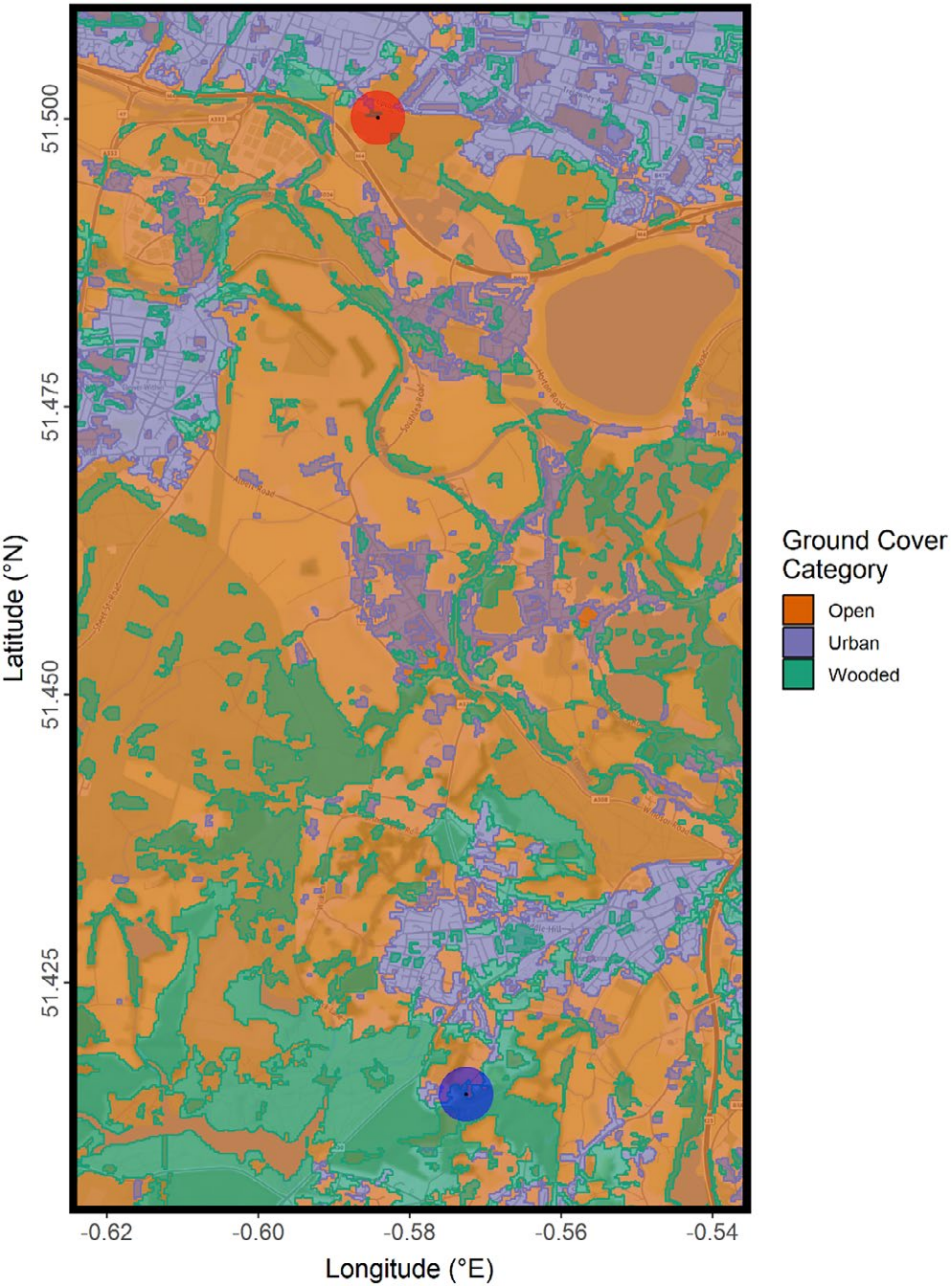
Surroundings of the experimental flight zone, coloured by polygonal habitat zones defining each category of ground cover assessed, based on Joint Nature Conservation Committee (JNCC) Phase 4 habitat categories, combined and exported using QGIS version 3.34.3. The red circular shaded area denotes the location of the experimental release site, from which birds were released, and the blue circular shaded area denotes that of the home site, where the birds were housed and homed to during experimental releases. Map created using *ggmap* version 4.0.0.

Three flocks of homing pigeons were kept in separate lofts (51.415368, −0.572615; Huntersdale, Royal Holloway University of London, GU25 4LW): 6 birds comprising flock L, 10 birds in flock N, and 8 birds in flock R. All birds were Jansen breed, comprising a homogenous mixture of colour morphs (predominantly blue bar, checker, and t‐check). Each individual bird was identified by numbered and coloured leg rings. The lofts had footprints of 3.6 × 2.4 m, with one‐way entrances that allowed the birds to enter the loft at will, but not escape. When inside, the birds had *ad libitum* access to food (Four Seasons Pigeon Corn, Johnston and Jeff) and water. The lofts were directly adjacent to each other, but the three flocks were kept physically and socially isolated at all times. The experimental flights involved repeated releases of all three flocks, allowing them to conduct homing flights from a common release site back to the lofts. The release site (51.500122, −0.584229; Upton Court Park, Slough, SL3 7LU) was approximately 9.58 km beeline north of the home site (Figure 1).

### 2.2 Biologgers and Body Mass

Each bird was equipped with a GPS logger and accelerometer (detailed below) for the provision of positional data and z‐axis body oscillations, respectively, and these were attached directly to the birds. Strips of the soft ‘loops’ side of Velcro were measured to fit both biologgers (detailed below) and adhered to the birds' backs using a minimal amount of two‐part epoxy (Araldite Rapid 2 × 15 mL). Particular care was taken to avoid any contact with bare skin, wing feathers, or tail feathers, and the corners of the strips were rounded to avoid any damage or discomfort to the bird. The feathers of the attachment zone were trimmed beforehand to minimise the prominence of the Velcro and to allow feathers to lay over it when not in use. Strips of the ‘hooks’ side of the Velcro were attached to the GPS and accelerometers, which allowed the loggers to be quickly and easily attached and removed.

The GPS loggers were custom‐modified for this study using the internal components of BT‐Q1300ST Sports Recorders (QStarz, product discontinued), compacted and encased in liquid electrical tape. The loggers were set to record at 5 Hz and weighed 14 g. Accelerometers were purchased online (Axivity AX3, 12.5–3200 Hz configurable, ±2–16gee configurable), with a recording rate of 100 Hz and weighing 11 g. Loggers were programmed, and data were downloaded daily using their respective proprietary software: OmGui for accelerometry, QTravel for GPS.

Additional mass loading in the form of biologgers can have significant effects on the behaviour and physical condition of flying birds (Bodey et al. 2018; Portugal and White 2018; Portugal et al. 2020), and it is commonly recommended that biologgers not exceed a combined 5% of the individual bird's mass due to compensatory mass loss (Kenward 2001). The birds used in this study were weighed on the day prior to commencing training flights. In 27 birds, the combined mass of biologgers constituted 4%–6% of body mass, in 13 birds, this was 6%–7%, and in one, it amounted to 7.81%. The strict enforcing of the 5% recommendation is primarily relevant when investigating long‐term flights—particularly that of migratory species—or the flights of small species (Thaxter et al. 2016; Portugal and White 2018, 2022); as such, it was decided that exceeding the limit by a few percent in the case of short and local flights of largely captive birds was not a cause for concern. The combined mean of the birds' masses was 436 g (±53 SD).

### 2.3 Experimental Flights

Each flock underwent 15 flights between 10 November 2022 and 11 December 2022, totalling 45 individual releases. Fifteen flights have previously been demonstrated to be sufficient for homing pigeon flocks to fully learn homing routes from a release site (Biro et al. 2004; Meade et al. 2005; Guilford and Biro 2014). On a given day of releases, each flock of birds was transferred to an individual carrier, with GPS loggers and accelerometers attached to each individual and set to record. The three carriers were then transported together by car to the release site (an approximately 20‐min journey) and placed on the ground facing the home site. The carrier of one flock (loft L) would be opened, and the birds allowed to fly free. After an interval of at least 10 min, this would be repeated for the second flock (loft R), and once more for the final flock (loft N). At the home site, the birds were observed until they had all returned inside their respective lofts to protect from potential predation. Loggers were retrieved, and the data downloaded either that evening or on the following morning.

There was a degree of data loss during the experimental period due to various issues, including technical errors or defects in the loggers (*N* = 9), the loss of loggers (*N* = 1), or birds themselves failing to return for unknown reasons (*N* = 9). A total of 41 flights were analysed (14 from lofts L and R and 13 from N), comprising 304 individual datasets.

### 2.4 Habitat Mapping and Data Processing

The mapping of ground cover category types in the flight corridor was done using GeoJSONs acquired from an online Department for Environment, Food, and Rural Affairs (DEFRA) Natural England habitat map, based on grouped Joint Nature Conservation Committee (JNCC) Phase 4 habitat definitions (Living England Habitat Map (Phase 4) 2023). Using QGIS version 3.34.3 (QGIS Geographic Information System 2023), the files of assorted habitat types as defined by Phase 4 were individually mapped and then grouped into the three categories of interest: Open ground (comprised of the Phase 4 categories Water; Acid, Calcareous, and Neutral Grassland; Arable and Horticultural; Bare Ground; Bare Sand; Bracken; Dwarf Shrub Heath; Fen, Marsh, and Swamp; Improved Grassland; Scrub; Unclassified), Wooded areas (Broadleaved, Mixed, and Yew Woodland; Coniferous Woodland), and Urban cover (Built‐up Areas and Gardens). These groups were each exported as GeoJSONs which could be plotted using the *ggmap* package version 4.0.0 (Kahle and Wickham 2013; Figure 1).

**FIGURE 2:**
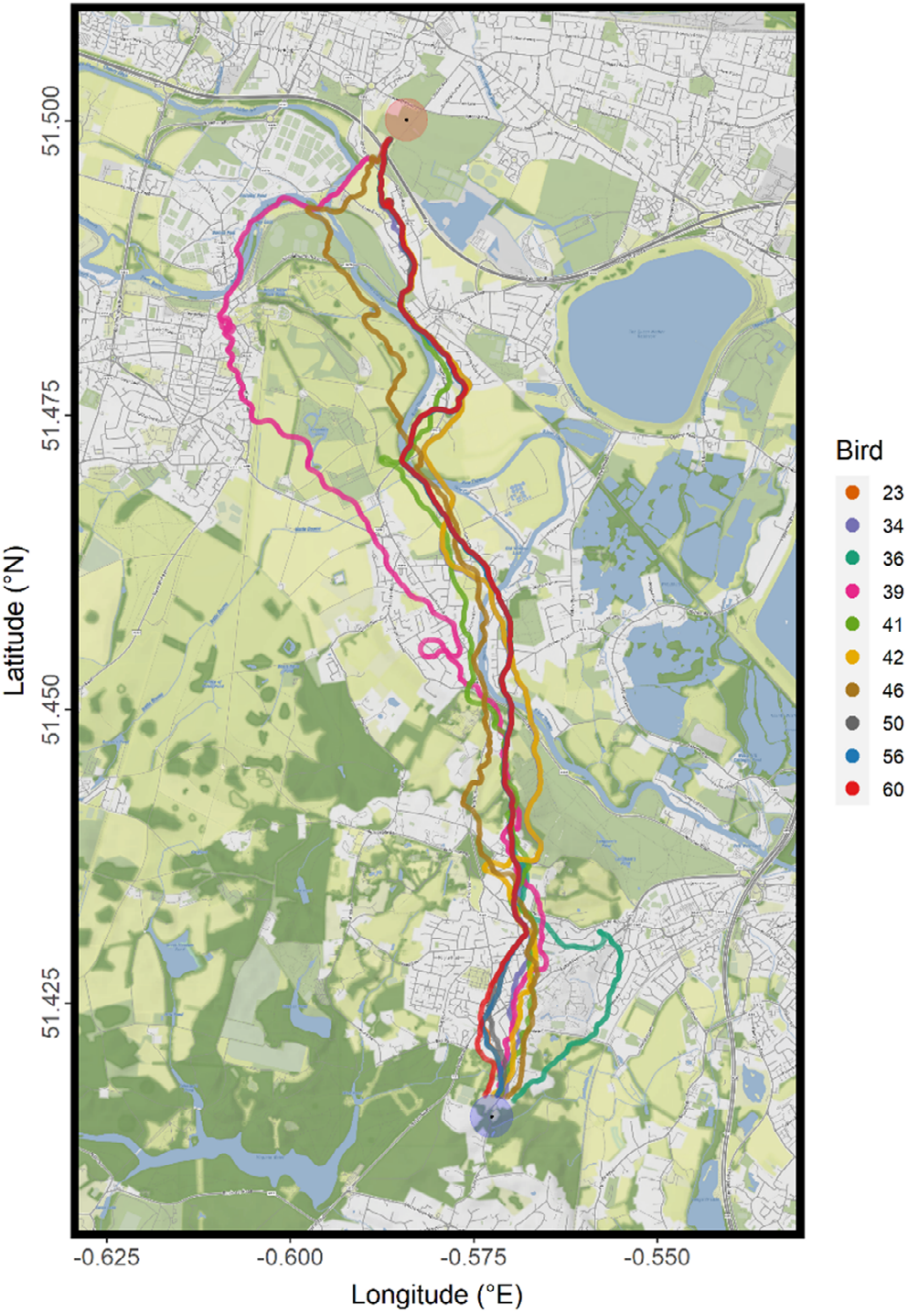
Flightpaths of Loft N birds during the release conducted on 10 November 2022, flying from the experimental release site to the home site. The red circular shaded area denotes the location of the experimental release site, from which birds were released during the experimental flights, and the blue circular shaded area denotes that of the home site, where the birds were housed and homed to during experimental releases. Both shaded areas visualise the approximate area data from within which was removed during analysis, to exclude atypical circling flight behaviour. Map created and data plotted using *ggmap* version 4.0.0.

The X‐axis, Y‐axis, and time readings of the GPS logger data sets were arranged into ‘XYT’ arrays, with each array constituting the full flight of one bird on a given date. These were converted to data frames which functioned as comprehensive spatial and temporal matrices that all other data could be mapped onto and allowed the easy plotting of flights and their associated data (Figure 2). All data were trimmed to remove any readings that fell within 200 m of the release and home sites, to exclude the atypical ascending, circling, and descending flight patterns at the beginning and ending of each flight (Taylor et al. 2017). The remaining timestamps were used as the range to which all data and variables were trimmed, including that based on accelerometry data.

To identify which category of ground cover birds were flying over at any given point, the XYT data frames were imported into the QGIS project containing the habitat category data. The datasets were clipped to each habitat category, recombined, and added back to the original XYT data frames. Every timestamp and positional reading had an associated habitat category level, which could be included as a variable in the analysis stage. A multinomial regression test from the R package *nnet* version 7.3‐19 (Venables and Ripley 2002) confirmed that Layer did not significantly correlate with either Bird or Loft, and these could, therefore, be used as random effects.

### 2.5 Flap Frequency and Autocorrelation

Flap frequencies were calculated using the procedure detailed in (Portugal et al. 2014) and used further in subsequent studies (Taylor et al. 2017; Sankey et al. 2019, 2021; Sankey and Portugal 2022, 2023). Vertical (z‐axis) readings of accelerometry data were used to first calculate the dorsal body amplitudes of the birds over the course of flight, with oscillations identifying individual wingbeats. External acceleration due to gravity, as well as velocity drift over extended periods, were filtered out using subtracted running means. Flap frequencies over every 15 data points were calculated and passed through a Butterworth filter (Shouran and Elgamli 2020). Due to the mismatch between the resolutions of the GPS and accelerometry data, made more complex by the calculation of flap frequencies per 15 accelerometry datapoints, it was decided to aggregate flap frequencies to the median values per second. The same was done to the GPS data sets, and the timestamps of both collections of data were subsequently aligned and merged. Data were subsampled at a rate of 15 points to account for autocorrelation, based on visual assessment of our flight data and the findings of prior study utilising similar data sets (Portugal et al. 2014; Sankey and Portugal 2019; Sankey et al. 2021). Although a degree of autocorrelation was found to have persisted, it was confirmed to be marginal and to have had no significant impact on the findings of our final models (see Appendix S1). To ensure timestamps matched within flock and flight data sets, the subsampling itself was done bird‐by‐bird for each loft and flight.

### 2.6 Time, Flock Dimensions, Flight Speed and Flock Position

Relatively fine‐scale individual positional readings recorded by the GPS loggers provided the data necessary to calculate all other variables included as predictors in the modelling process. These variables were calculated from the full data sets and either subsampled as part of the autocorrelation removal described in Section 2.5, or merged by timestamp and positional readings to the subsampled data set for final analysis. Given that timestamps were in raw UTC format, an alternative measure of flight time was necessary to identify changes over the course of individual flights. We added an increasing numeric index aligned with flight time that acted as a measure for the length of the flight in seconds, which reset at the start of every flight (starting at 0 s transpired). A similar index was created that increased with ascending flight date to represent incremental increases in the familiarity of the flight zone and experience of birds over multiple flights (Taylor et al. 2017). We refer to these indices as ‘flight time’ and ‘iteration’, respectively.

Prior to subsampling, pairwise comparison of individual positions identified which birds were flying within 10 m of each other—these were considered as flying cohesively in a flock (Yomosa et al. 2015; Kano et al. 2021; Sankey and Portugal 2023). On occasion, flocks would divide into one or more sub‐flocks, the data for which we did not include in our analysis. To account for splits, birds which were 40 m or further from the calculated centroid (see below) at any given timestamp were excluded, and the centroid re‐calculated for that timestamp. This method of centroid calculation ensured that when flock division occurred, the larger group would have a greater weighted influence on flock centroid, and subsequently, only that group's data would be retained for analysis. This also allowed for birds to reintegrate with their flocks and their data to still be included in our analysis, rather than discarding all subsequent data of any bird that at any point during the flight was further than 40 m from the centroid. The same pairwise comparison enabled the calculation of the approximate centroid of each flock across every flight's timestamp, based on the procedure described in Sankey and Portugal (2019). From stepwise changes in flock centroid, the direction of travel of the flock was identified. From this, the flock's axes, parallel and perpendicular to the direction of travel, could be ascertained at every timestamp.

The three flock dimension variables were then calculated at every timestamp. Absolute flock spread was defined and calculated as the distance in metres between the frontmost and backmost members of the flock added to the distance between the leftmost and rightmost members. This provided an overall metric for how spread out the birds of a flock were, given a constant number of birds flocking together. Group size, as we define it here, was the number of individuals in a flock that continued to fly cohesively in their groups following initial release, as determined by the flock cohesion filtering process described above. This provided a much more accurate measure of the number of birds that flocked together than simply the number of birds being released, as often individual birds departed their flocks to fly alone or in smaller groups (see, e.g., in Figure 2). Individual distance to the centroid was taken as each bird's direct distance to the flock centroid, measured in metres, providing an alternative and more detailed measure of how cohesively a flock's birds were flying. Individual birds' flight speeds were calculated from changes in GPS position over every datapoint, also providing a measure of distance travelled over time. Identifying the direction of flock travel also enabled the determination of each bird's position within the flock relative to the centroid, which was measured in three variables: (1) left–right distance, being the distance in metres of an individual bird from the centroid perpendicular to the direction of travel (positive values to the right of the centre, negative to the left); (2) front‐back distance, the distance of an individual to the centroid parallel to the direction of travel (positive values ahead of the centre line, negative behind it); and (3) position, a binary reading of whether a bird was in front or behind the centroid, which was not modelled as a dependent variable due to its categorical nature.

### 2.7 Modelling

To determine how the variables (flap frequency, absolute flock spread, distance to centroid, group size, flight speed, front‐back distance, and left–right distance) interacted with ground cover category and temporal changes (flight time and iteration), we utilised a linear mixed effect regression approach. Data from all variables fitted normal distributions except for absolute flock spread and distance to centroid, which were subsequently square root‐transformed.

For each modelled variable, a ‘full’ version of its model's syntax was written that included ground cover category and every calculated variable (described in Section 2.5 and 2.6, including those which were themselves being modelled, where applicable), as well as flight time, iteration, and their respective interactions with ground cover category. The fixed effects of ‘Bird’ and ‘Loft’ were used as random variables for every model. Variance inflation factor (VIF) from *car* version 3.1.1 (Fox and Weisberg 2019) was used to assess the severity of multi‐collinearity in the variables of these full‐variable model syntaxes, and any collinear variables were subsequently removed, providing the final ‘verified’ full model syntaxes. From these verified full syntaxes, every possible combination of variables was run using *lme4* version 1.1‐35.1 (Bates et al. 2025), and their Akaike Information Criterion (AIC) scores calculated (Akaike 2011). The syntaxes of delta‐AIC < 2 from the lowest were identified, and from these, the model of best fit was selected by whichever had the highest *r* ^2^ value (Lewis‐Beck and Lewis‐Beck 2015). Where *r* ^2^ values matched to the nearest fifth decimal place, the simplest model containing the fewest variables was chosen. Outlier values were removed using Cook's Distances (Cook 1977), and the models were re‐run, providing the final outputs presented below. All analyses and data management were done in R version 4.2.1 (R Core Team 2022), and figures were created using *ggplot2* version 3.5.1 (Wickham 2016).

## 3 Results

### 3.1 Changes Over Ground Cover

**FIGURE 3:**
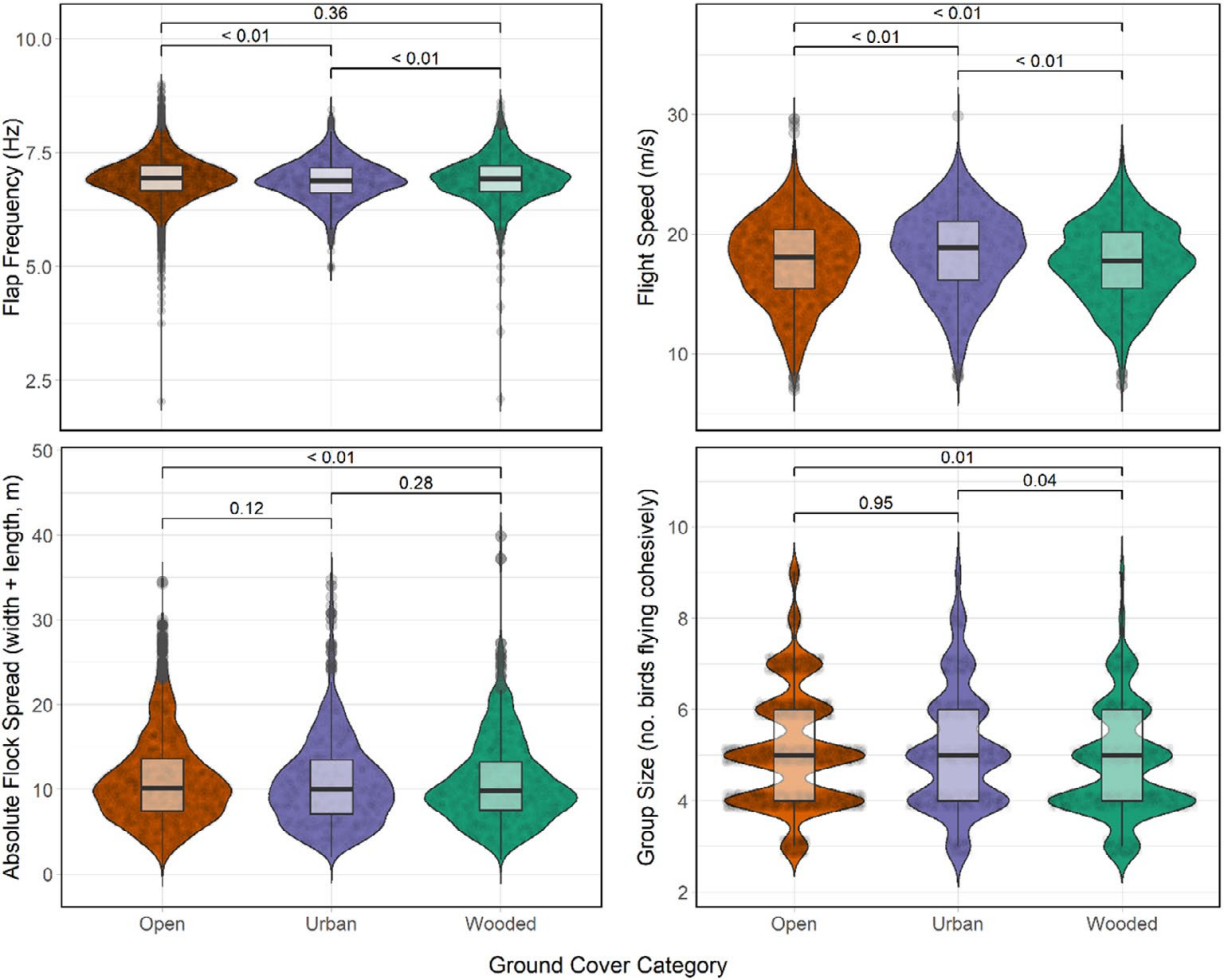
Four flight and flocking variables of homing pigeon flocks identified as being significantly influenced by ground cover habitat category. Model outliers were removed via Cook's distance, and *p* values of pairwise differences are listed above each pair. Significance boundary was *p* < 0.05. Plotting was done using *ggplot2* version 3.5.1.

**FIGURE 4:**
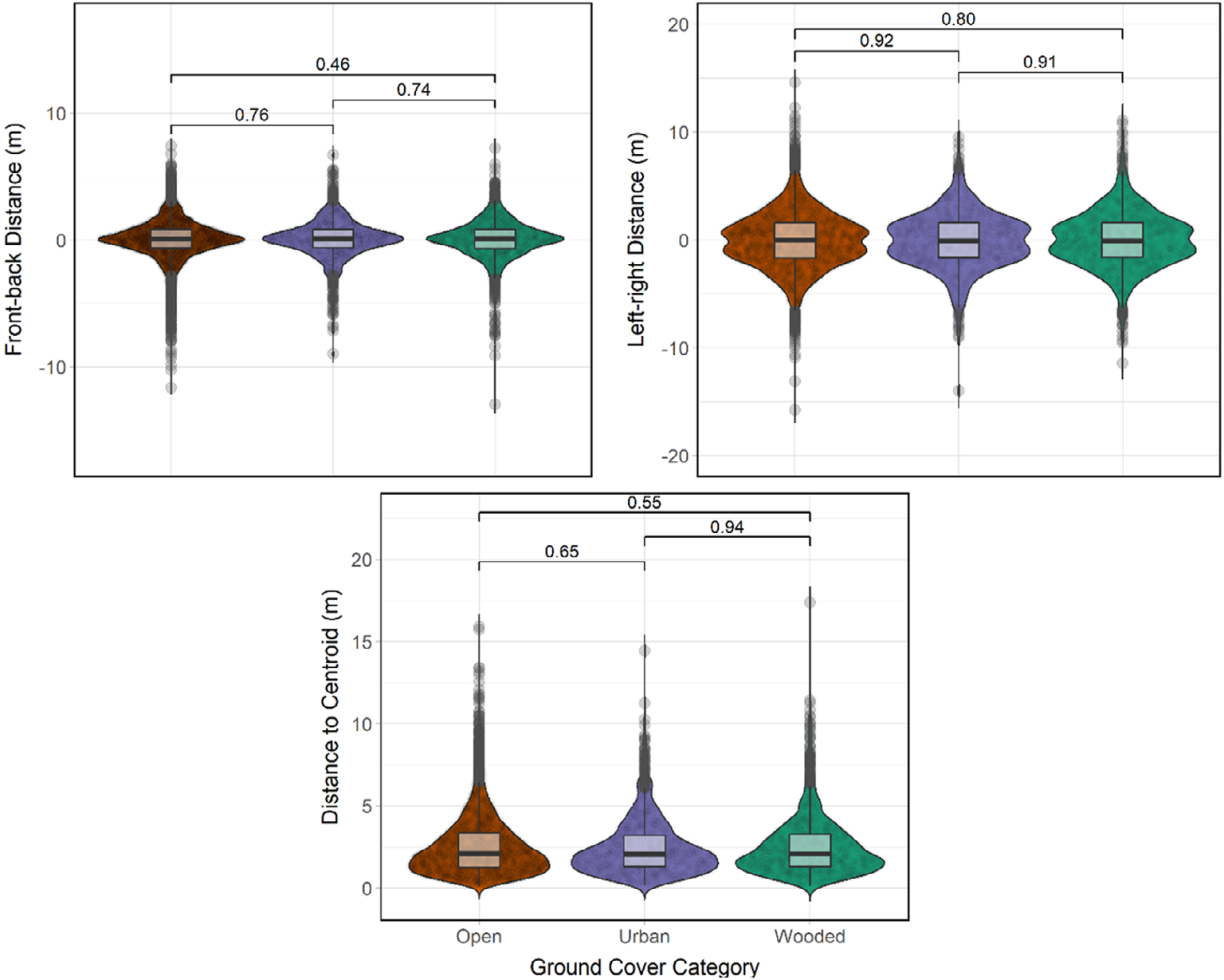
Flight and flocking variables of homing pigeon flocks identified as not being significantly influenced by ground cover habitat category. Model outliers were removed via Cook's distance, and *p* values of pairwise differences are listed above each pair. Significance boundary was *p* < 0.05. Plotting was done using *ggplot2* version 3.5.1.

Four models from the model selection process retained Layer as an influential variable: flap frequency, absolute flock spread (the additive distance between the frontmost and backmost and leftmost and rightmost birds in a flock), group size (the number of birds flying cohesively at any given time after initial release), and flight speed (Table 1, Figure 3). None of the models of front‐back or left–right distance passed non‐singularity, and the model of best fit for distance to centroid did not retain habitat category type as a significant determinant (Figure 4).

**TABLE 1:** Summaries of ground cover category, flight time and flight iteration‐related independent variables retained in the linear mixed effects regression models of best‐fit describing changes in flap frequency, absolute flock spread, group size and flight speed over different ground cover habitat categories.

Response var.	Independent var.	Estimate	SE	*F*	*p*
Flap frequency (*N* = 6999, AIC = 8429.52, *r* ^2^ = 0.06) **[Open Cover]**	**Urban ground cover**	**−0.10**	**0.03**	**6.40**	**< 0.01**
	Wooded ground cover	0.03	0.03	6.40	0.36
	**Flight time**	**−0.14**	**0.01**	**182.76**	**< 0.01**
	Flight iteration	< −0.01	< 0.01	4.88	0.79
	Urban cover over time	−0.01	0.02	1.42	0.56
	Wooded cover over time	0.03	0.02	1.42	0.16
	**Urban cover over iteration**	**0.01**	**< 0.01**	**3.97**	**< 0.01**
	Wooded cover over iteration	< 0.01	< 0.01	3.97	0.58
**[Wooded Cover]**	**Urban ground cover**	**−0.12**	**0.04**	**6.40**	**< 0.01**
	Urban cover over time	−0.04	0.02	1.42	0.11
	Urban cover over iteration	0.01	< 0.01	3.97	0.07
Absolute flock spread (square root‐transformed) (*N* = 6800, AIC = 11638.22, *r* ^2^ = 0.45) **[Open Cover]**	Urban ground cover	0.06	0.04	5.13	0.12
	**Wooded ground cover**	**0.11**	**0.04**	**5.13**	**< 0.01**
	Flight time	−0.01	0.01	0.01	0.32
	**Flight iteration**	**−0.01**	**< 0.01**	**67.19**	**< 0.01**
	Urban cover over time	0.04	0.03	0.97	0.16
	Wooded cover over time	0.01	0.03	0.97	0.72
	Urban cover over iteration	−0.01	< 0.01	3.97	0.08
	**Wooded cover over iteration**	**−0.01**	**< 0.01**	**3.97**	**0.01**
**[Wooded Cover]**	Urban ground cover	−0.05	0.05	5.13	0.28
	Urban cover over time	0.03	0.03	0.97	0.41
	Urban cover over iteration	< 0.01	0.01	3.97	0.59
Group size (*N* = 6615, AIC = 18273.04, *r* ^2^ = 0.23) **[Open Cover]**	Urban ground cover	< −0.01	0.07	3.79	0.95
	**Wooded ground cover**	**−0.17**	**0.06**	**3.79**	**0.01**
	**Flight time**	**−0.62**	**0.02**	**736.85**	**< 0.01**
	**Flight iteration**	**−0.02**	**< 0.01**	**14.44**	**< 0.01**
	Urban cover over time	0.03	0.04	5.70	0.52
	**Wooded cover over time**	**0.15**	**0.05**	**5.70**	**< 0.01**
	Urban cover over iteration	0.01	0.01	3.44	0.15
	**Wooded cover over iteration**	**0.02**	**0.01**	**3.44**	**0.01**
**[Wooded Cover]**	**Urban ground cover**	**0.17**	**0.08**	**3.79**	**0.04**
	**Urban cover over time**	**−0.13**	**0.06**	**5.70**	**0.02**
	Urban cover over iteration	−0.01	0.01	3.44	0.46
Flight speed (*N* = 6696, AIC = 35686.15, *r* ^2^ = 0.02) **[Open Cover]**	**Urban ground cover**	**0.69**	**0.23**	**11.58**	**< 0.01**
	**Wooded ground cover**	**−0.67**	**0.22**	**11.58**	**< 0.01**
	Flight iteration	0.02	0.01	10.17	0.22
	Urban cover over iteration	−0.01	0.03	4.85	0.81
	**Wooded cover over iteration**	**0.08**	**0.03**	**4.85**	**< 0.01**
**[Wooded Cover]**	**Urban ground cover**	**1.36**	**0.28**	**11.58**	**< 0.01**
	**Urban cover over iteration**	**−0.09**	**0.03**	**4.85**	**0.01**

*Note:* Significant variables highlighted in bold. Ground cover category listed in bold in square brackets is the reference level of the respective modelled independent variables.

**FIGURE 5:**
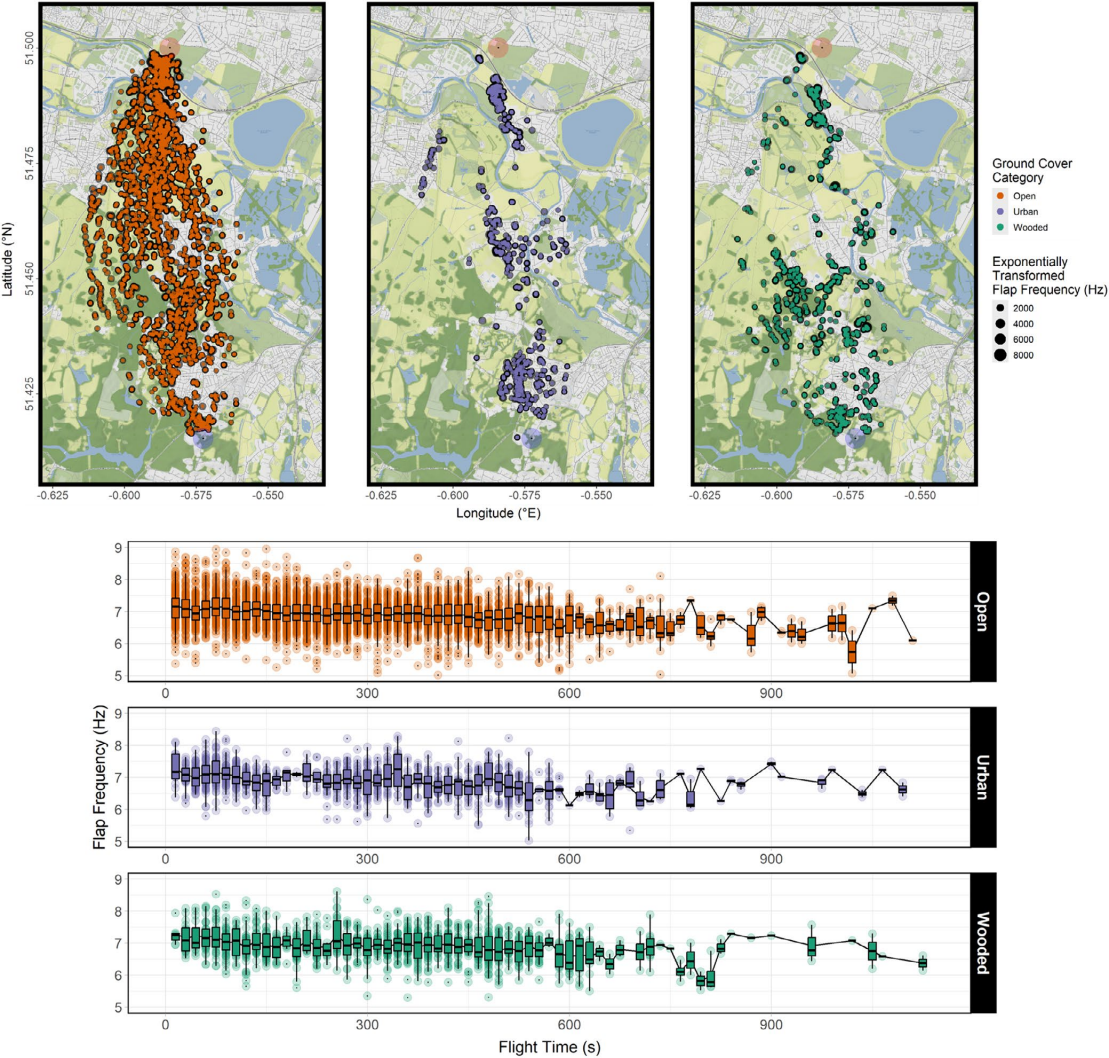
Combined flap frequency readings of three flocks of homing pigeons over 14 flights mapped over the flight corridor (top), and plotted over flight time (bottom). Data are post‐subsampling and ‐outlier removal using Cook's distances based on a GLMM of best fit. Map flap frequencies are exponentially transformed, and the plot over flight time excludes 22 datapoints for visualisation. Maps were created using *ggmap* version 4.0.0, and plots created using *ggplot2* version 3.5.1.

**FIGURE 6:**
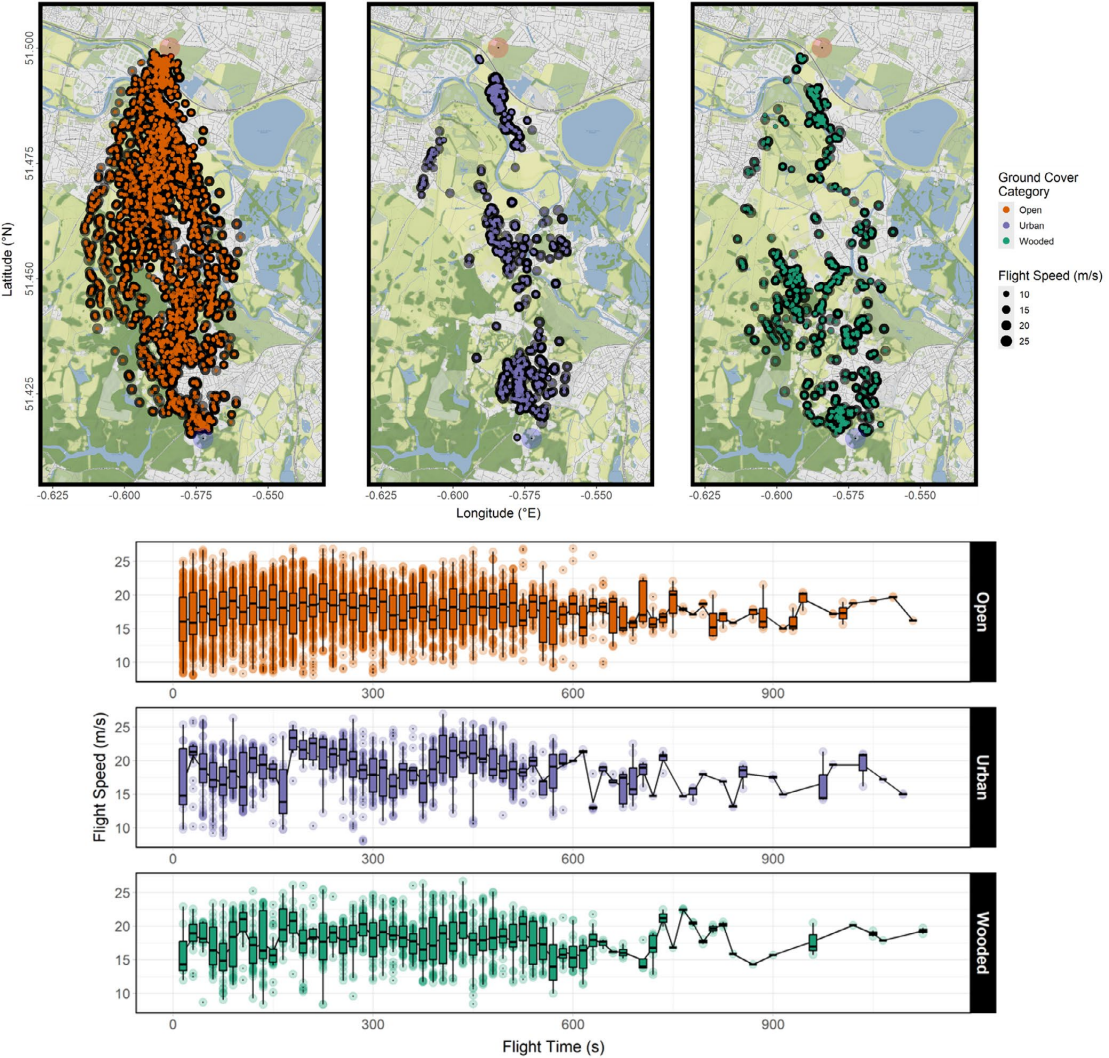
Combined flight speed readings of three flocks of homing pigeons over 14 flights mapped over the flight corridor (top), and plotted over flight time (bottom). Data are post‐subsampling and ‐outlier removal using Cook's distances based on a GLMM of best fit. The plot over flight time excludes 25 datapoints for visualisation. Maps were created using *ggmap* version 4.0.0 and plots created using *ggplot2* version 3.5.1.

**FIGURE 7:**
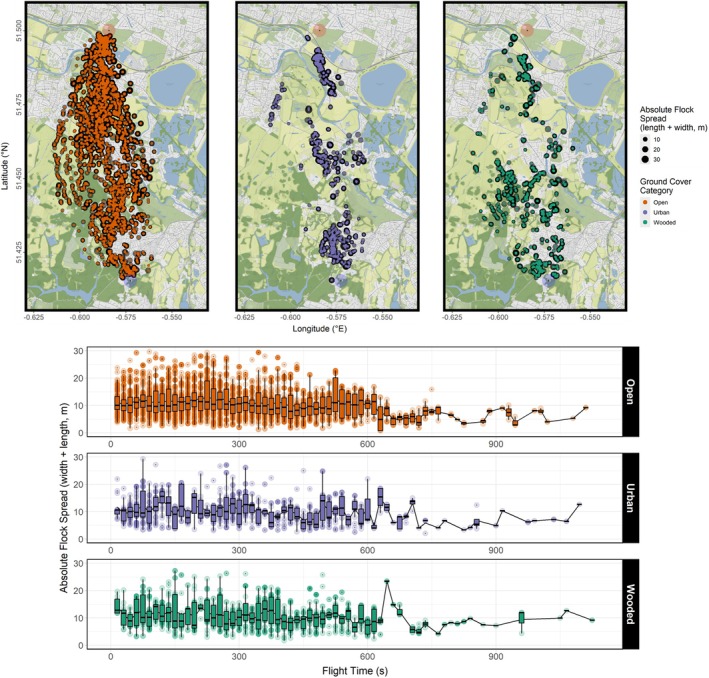
Combined absolute flock spread readings of three flocks of homing pigeons over 14 flights mapped over the flight corridor (top), and plotted over flight time (bottom). Data are post‐subsampling and ‐outlier removal using Cook's distances based on a GLMM of best‐fit. The plot over flight time excludes 21 datapoints for visualisation. Maps were created using *ggmap* version 4.0.0, and plots created using *ggplot2* version 3.5.1.

**FIGURE 8:**
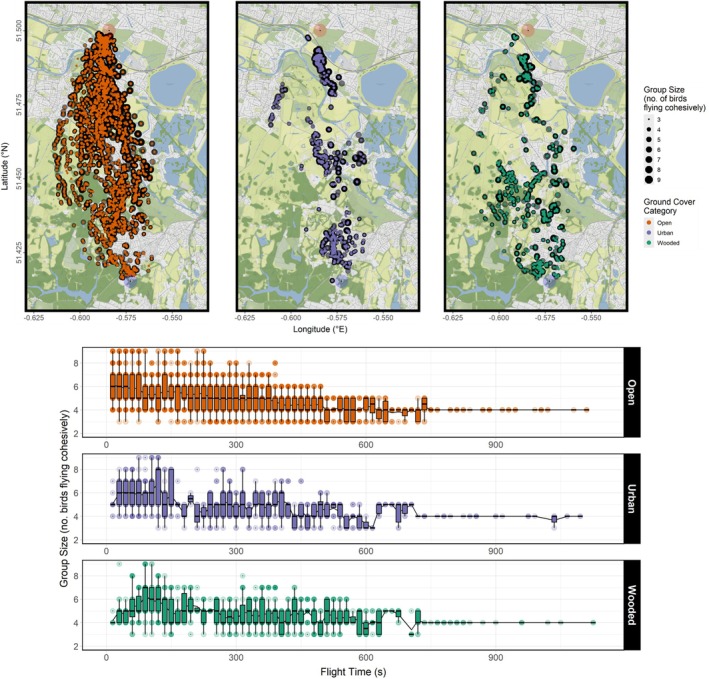
Combined group size readings of three flocks of homing pigeons over 14 flights mapped over the flight corridor (top), and plotted over flight time (bottom). Data are post‐subsampling and ‐outlier removal using Cook's distances based on a GLMM of best fit. Maps were created using *ggmap* version 4.0.0, and plots created using *ggplot2* version 3.5.1.

At least one independent layer effect was found to be significant in all four models. Individual birds reduced their flap frequencies but increased flight speed when flying over urban areas (Figures 3, 5, and 6) relative to open ground, and flight speed was reduced when flying over wooded areas (Figures 3 and 6). Flocks collectively increased their flock spread (Figures 3 and 7) and reduced group size (Figures 3 and 8) that is, fewer birds continued to fly cohesively and did so less densely when flying over wooded areas.

### 3.2 Interaction and Temporal Effects

Interaction effects between levels of habitat category and flight time or iteration were retained in all four significant models. The magnitude of the effect flying over wooded areas had on absolute flock spread, group size, and flight speed decreased with increasing flight iteration (*β* = −0.01, *F* = 3.97, *p* = 0.01; *β* = 0.02, *F* = 3.44, *p* = 0.01; *β* = 0.08, *F* = 4.85, *p* < 0.01), and the same was true for the effect flying over urban areas had on flap frequency (*β* = 0.01, *F* = 3.97, *p* < 0.01). Flight time‐based changes to habitat category‐related changes were retained only in the model of group size, where the negative trend that was observed over wooded areas weakened the later into a flight the birds were (*β* = 0.15, *F* = 5.70, *p* < 0.01).

While there was no interaction effect between flight time and habitat category‐related changes, flap frequency did reduce significantly over the course of flight time (*β* = −0.14, *F* = 182.76, *p* < 0.01). There was no significant correlation between flap frequency and flight iteration independent of the habitat category‐related changes. Absolute flock spread did significantly decrease over flight iteration (*β* = −0.01, *F* = 67.19, *p* < 0.01), and the number of birds flying cohesively was found to significantly reduce both over the course of individual flights (*β* = −0.62, *F* = 736.85, *p* < 0.01) and over iterative flights (*β* = −0.02, *F* = 14.44, *p* < 0.01). Despite the interaction effect describing a reduced effect size in the correlation between reduced flight speed over wooded areas, there was no significant correlation between flight speed and flight iteration itself. All models which passed non‐singularity, including that of distance to centroid (AIC = 9276.26, *r* ^2^ = 0.04), retained multiple additional variables describing significant changes (Table 2).

**TABLE 2:** Output summaries of all significant variables retained in linear mixed effects regression models for flap frequency, absolute flock spread, distance to centroid, group size and flight speed.

Response var.	Independent var.	Estimate	SE	*F*	*p*
Flap frequency (*N* = 6999, AIC = 8429.52, *r* ^2^ = 0.06) **[Open Cover]**	Urban ground cover	−0.10	0.03	6.40	< 0.01
	Flight time	−0.14	0.01	182.76	< 0.01
	Distance to centroid	−0.02	< 0.01	28.40	< 0.01
	Group size	0.04	0.01	61.69	< 0.01
	Flight speed	−0.01	< 0.01	13.30	< 0.01
	Front‐back distance	−0.01	< 0.01	5.83	0.02
	Urban cover over iteration	0.01	< 0.01	3.97	< 0.01
**[Wooded Cover]**	Urban ground cover	−0.12	0.04	6.40	< 0.01
Absolute flock spread (square root‐transformed) (*N* = 6800, AIC = 11,638.22, *r* ^2^ = 0.45) **[Open Cover]**	Wooded ground cover	0.11	0.04	5.13	< 0.01
	Flight iteration	−0.01	< 0.01	67.19	< 0.01
	Distance to centroid	0.22	< 0.01	2851.65	< 0.01
	Group size	0.20	0.01	918.66	< 0.01
	Flight speed	−0.01	< 0.01	7.32	0.01
	Front‐back distance	0.05	0.01	71.12	< 0.01
	Front‐positioned	0.06	0.02	8.78	< 0.01
	Wooded cover over iteration	−0.01	< 0.01	3.97	0.01
Distance to Centroid (square root‐transformed) (*N* = 6386, AIC = 9276.26, *r* ^2^ = 0.04) **[Open Cover]**	Flight iteration	−0.01	< 0.01	20.39	< 0.01
	Flap frequency	−0.06	0.01	18.44	< 0.01
	Front‐back distance	−0.06	0.01	138.68	< 0.01
Group size (*N* = 6615, AIC = 18,273.04, *r* ^2^ = 0.23) **[Open Cover]**	Wooded ground cover	−0.17	0.06	3.79	0.01
	Flight time	−0.62	0.02	736.85	< 0.01
	Flight iteration	−0.02	< 0.01	14.44	< 0.01
	Absolute flock spread	0.08	< 0.01	874.54	< 0.01
	Distance to centroid	−0.06	0.01	56.05	< 0.01
	Flap frequency	0.16	0.03	37.92	< 0.01
	Flight speed	−0.02	< 0.01	20.12	< 0.01
	Wooded cover over time	0.15	0.05	5.70	< 0.01
	Wooded cover over iteration	0.02	0.01	3.44	0.01
**[Wooded Cover]**	Urban ground cover	0.17	0.08	3.79	0.04
	Urban cover over time	−0.13	0.06	5.70	0.02
Flight speed (*N* = 6696, AIC = 35686.15, *r* ^2^ = 0.02) **[Open Cover]**	Urban ground cover	0.69	0.23	11.58	< 0.01
	Wooded ground cover	−0.67	0.22	11.58	< 0.01
	Absolute flock spread	−0.03	0.01	8.34	< 0.01
	Group size	−0.13	0.04	10.86	< 0.01
	Flap frequency	−0.33	0.09	12.01	< 0.01
	Front‐back distance	0.08	0.04	5.46	0.02
	Wooded cover over iteration	0.08	0.03	4.85	< 0.01
**[Wooded Cover]**	Urban ground cover	1.36	0.28	11.58	< 0.01
	Urban cover over iteration	−0.09	0.03	4.85	0.01

*Note:* Ground cover category listed in bold in square brackets is the reference level of the respective modeled independent variables.

## 4 Discussion

### 4.1 Significant Trends and Our Predictions

We identified four variables describing the flight and flocking patterns of homing pigeons which significantly correlated with changes to the ground cover over which the birds were flying. Our findings regarding changing flight speed over ground cover concur with that of Garde et al. (2021), who reported a significant but small reduction in speed over woodland. However, whereas Garde et al. (2021) found that their birds reduced speed by approximately 0.17 m/s when flying over wooded areas, our birds exhibited much larger changes. Our models found that when flying over woodland, the birds slowed by approximately 0.67 m/s relative to their speed over open areas, and over urban ground we identified a near‐perfect mirroring of this change, with birds speeding up by 0.69 m/s relative to open ground. These each represent approximately 3.8% changes relative to the mean flight speed across all habitats (17.92 m/s) and directly oppose our expectation that birds would speed up to minimise time spent over woodland.

Models of our directional measures of individual spatial positioning within a flock (front‐back and left–right distance) failed to pass singularity. Singularity is a common issue faced by linear regressions, without a single universal solution (Klein and Nakamura 1962; Takada et al. 1995; Haines and Fiori 2013; Dwivedi et al. 2020). For the purposes of this study, we took singularity of flock position models as an indication that we should accept our fourth hypothesis, rejecting the significance of ground cover to individual positioning within flocks.

An explanation for reduced flight speed over woodland, posited by Garde et al. (2021), was that it might be indicative of the adoption of more focused anti‐predator flight behaviours in light of heightened perceived predator threat. Prey species are often observed to undergo sudden, sharp turns as an effective method of evading predatory bird species such as Eurasian goshawks ( *Accipiter gentilis* ; Hedenström and Rosén 2001; Su et al. 2012; Kane et al. 2015; Papadopoulou et al. 2022a). Highly tortuous flight, such as that adopted during predator evasion, is associated with higher flap frequencies (Usherwood et al. 2011; Ling et al. 2018); however, we did not see this in our birds. Although we recorded an increase in flap frequency when flying over woodland, it was an order of magnitude smaller than the change seen when flying over urban space (an increase of 0.03 Hz vs. a reduction of 0.10 Hz) and statistically non‐significant. Contrary to our expectations, this initially presents an image of homing pigeons adopting a more ‘relaxed’ flight posture when flying over woodland than when in open or urban areas (see below). Alternatively, it may indicate the prioritisation of slower, more stable movement in line with an increased wariness of potential predators (Howland 1974), as seen in some fish (Ryer et al. 2004; Johansson and Andersson 2009; Jones and Godin 2009), reptile (Punzo 2007), and mammal species (Blumstein et al. 2004; West et al. 2018). To our knowledge, in line with Garde et al.'s (2021) conclusions, this is a novel finding in the study of transitory bird flight in general (due primarily to a lack of study in other species).

The approach we took to this study was based on the idea that individual birds alter their behaviour in response to perceived threats or benefits associated with certain habitat types (Hamm and Shettleworth 1987; Whittingham and Evans 2004; Carrascal and Alonso 2006; Cresswell 2008; Garde et al. 2021; Clayton et al. 2022). We predicted that due to the prevalence of predator species in woodland areas (Götmark and Post 1997; Dixon 2002; Henderson et al. 2004), and the resemblance of urban built‐up areas to natural rock dove roosting habitats (Lundholm and Richardson 2010; Ferretti 2019), homing pigeon flocks would display ‘stressed’ and ‘relaxed’ adjustments respectively. We expected flock density readings and flap frequencies to increase over wooded terrain as a signal of pre‐emptive predator response (Cresswell 1994; Quinn and Cresswell 2006; Usherwood et al. 2011; Sankey and Portugal 2019; Papadopoulou et al. 2022a), and possibly increased flight speed to minimise the time spent flying over ‘dangerous’ terrain (Garde et al. 2021). Our data conclusively refute these predictions, as in all flight variables found to be significantly affected by ground cover, the effects were opposite to what we expected to find.

The absence of increased predator defence over wooded terrain may relate to the nature of homing pigeon flocks in general. Papadopoulou et al. (2022a) point out that pigeon flocks (particularly smaller ones) tend to be low density and not particularly effective in terms of predator confusion compared with species such as common starlings (Landeau and Terborgh 1986; Hogan et al. 2017). While pigeons will undergo high‐tortuosity flock splits even at high distances from a perceived threat, this appears to be an exclusively reactionary behaviour rather than an environmentally driven pre‐emptive decision (Papadopoulou et al. 2022a). Sankey et al. (2021) report that homing pigeons do not act according to the expectations of traditional selfish‐herd behaviour, and this is in line with our findings regarding our measures of flock dimensions. Most prey animals under threat by predators (even if only perceived, not actual) are expected to prefer a central position, with groups generally concentrating together into higher density formations (Viscido and Wethey 2002; Lee et al. 2006; De Vos and O'Riain 2010; Aplin et al. 2014). We observed no significant change in an individual's proximity to the centroid over any ground cover types, and in fact the flocks appeared to spread out more when flying over woodland, although this change was of minor significance (an expansion of approximately 5 cm in every direction) and may have been counteracted by the high risk of interindividual collisions during flight.

Although the notion of homing pigeons innately exhibiting low levels of predator‐defence behaviour might explain the absence of defensive behaviours, it does not explain why the birds appeared to exhibit ‘relaxed’ flight behaviour when above woodland, particularly the significant reduction in flight speed (discussed further below). Nor does it explain the contrasting low‐energy but high‐speed flight we observed over urban terrain. Our homing pigeons decreased their flap frequencies over urban spaces by approximately 0.1 Hz (0.12 Hz compared with woodland). Although this frequency reduction only represents a 1.44% change from the mean of 6.93 Hz, it is comparable to a tenfold reduction in flock density (Usherwood et al. 2011), a 10‐m increase in distance to the nearest conspecific (Taylor et al. 2019), or the equivalent of 5 g of mass being removed (Sankey and Portugal 2023). Low flap frequency combined with high flight speed principally describes an overall trend of gliding, possibly descending, flight (Dial 1992; Muijres et al. 2012; Norberg 2013; Sachs 2015, 2017; Garde et al. 2021). Gliding flight, although significantly more energetically efficient than powered flight (Dial 1992; Norberg 2013; Portugal et al. 2014; Sachs 2015, 2017; Usherwood 2016), is incongruent with the predator defence tactics utilised by globular flocks (Papadopoulou et al. 2022a, 2022b).

### 4.2 Aerodynamics Over Different Landscapes

One environmental parameter which is likely to be significantly influencing how flight behaviours change over ground cover is the aerodynamics at play in the air above the different habitats. Wind speed and direction are known to significantly affect the way flying species move through environments, and particular study into this has been done regarding migratory bird species (Hedenström et al. 2002; Thorup et al. 2003; Gibb et al. 2017; Bruderer et al. 2025). The greatest challenge in determining how the aerodynamic conditions in areas above different environmental covers affect flight behaviour lies in obtaining fine‐scale measures of wind movement. Studies of migratory species have generally used large‐scale meteorological data, or historical trends, that are not suitable for the extreme‐resolution data involved in behavioural analysis (Evans 1966; Vähätalo et al. 2004; Gibb et al. 2017).

Our birds typically flew between 12 and 280 m above ground (see Appendix S1), and other studies have reported flight altitudes of between 10 and 500 m (Adams et al. 1999) or 50–250 m (Zaleshina and Zaleshin 2020). Aerodynamic variables, in particular wind speed and direction, vary drastically at the fine scale and at different heights, temperatures, times of day, times of year, and the specific land features being flown over (McCutchan and Fox 1986; Tar 2008; Bañuelos‐Ruedas et al. 2010; Fang et al. 2018; Wu et al. 2018; Solano et al. 2021). We could speculate, for example, that our birds demonstrated a higher propensity for gliding flight over urban areas because there was a greater prevalence of thermal updrafts (Hildebrand and Ackerman 1984), necessitating fewer and shorter periods of powered flight (Pennycuick 1972; Leshem and Yom‐Tov 1996; Ákos et al. 2010). There have been investigations that demonstrate how the surface ‘roughness’ (comparable to the density of visual cues discussed below) of woodland and urban areas may cause wind speeds to reduce (Grimmond et al. 1998; Grimmond and Oke 1999; Kent et al. 2017), but these are also highly variable at fine scales.

Attempts to ascertain likely causes of behavioural change are further complicated by evidence that changes in flight behaviour responding to wind direction, speed, etc. vary on an individual and case‐by‐case basis (Alerstam 1978; Green and Alerstam 2002; Thorup et al. 2003; Liechti 2006). Liechti (2006) provides a comprehensive review of the role that individualism and environmental context play in flight behaviour decision‐making, as well as the challenges in attempting to investigate this generally. Individual wind‐speed or air‐condition loggers carried by the birds, such as backpack‐mounted barometers or wing‐feather mechanoreceptors, may be the answer: these can be used in conjunction with GPS and/or accelerometry to better understand how individuals respond to changing weather conditions (Brown and Fedde 1993; Liechti 2006). However, any solution that necessitates the addition of a new biologgers will come up against the challenge of maximum mass limits (Portugal et al. 2020; Portugal and White 2022). There seems to be a notable lack of research into how we may better understand the role that wind speed and other relevant meteorological changes will affect behaviour at the fine scale, most continuing to focus on large‐scale changes (Fang et al. 2018; Mainwaring et al. 2021; Thorne et al. 2023; Davies et al. 2024; Bruderer et al. 2025). We argue that high‐detail tracking of individual movements may offer an effective method for assessing changes in response to environmental conditions going forward, discussed further below. Alternatively, studies of this kind could be conducted during the winter, as the movement and turbidity of parallel winds have been demonstrated to be significantly impacted by the presence of canopies that are in leaf (Shepard et al. 2016).

### 4.3 Landmarks, Familiarity and Navigation

Landmarks likely play a role in determining the homing behaviour and speed of birds. The use of natural or man‐made landmarks in identifying familiar landscapes and in aiding navigation has been demonstrated in a number of species, including homing pigeons (Burt et al. 1997; Kamil et al. 2001; Bingman and Cheng 2005; Sovrano et al. 2005; Vyssotski et al. 2009; Chan et al. 2012; Mora et al. 2012; Zaleshina and Zaleshin 2018; Wiltschko and Wiltschko 2023). Some evidence suggests proximal environmental features and cues may take precedence over the more often‐highlighted use of distal landmarks for navigation (Etienne et al. 1995; Yaski and Eilam 2007).

Most recent research into the relationship between landmark‐based route familiarity and flight behaviour has focused on changes in route efficiency (Biro et al. 2004, 2006; Meade et al. 2006), rather than flight speed. However, Holland (2003) and Guilford and Biro (2014) provide a comprehensive review of evidence that demonstrates visual familiarity with an area will improve the homing speed of birds flying through it (Braithwaite 1993; Braithwaite and Newman 1994; Braithwaite and Guilford 1995; Braithwaite et al. 1997; Burt et al. 1997; Gagliardo et al. 2001; Biro et al. 2002). Mann et al. (2014) carried out a similar experimental procedure to ours, combined with high‐detail land cover height texture maps, with the aim of identifying how the topography and density of visual information (i.e., landmarks) may affect the development of visual familiarity. Mann et al.'s (2014) findings, and that of the preceding study into route‐learning in urban spaces (Wiltschko et al. 2007; Mann et al. 2010; Schiffner et al. 2013), support their suggestion that regions with high amounts of visual noise, namely dense woodland and highly built‐up areas, result in the birds having a reduced ability (or preference) to memorise routes through these spaces. High visual noise resulting in poor route learning and reduced familiarity, combined with the findings compiled by Holland (2003) and Guilford and Biro (2014) regarding flight speed and route familiarity, could explain the significant reduction in speed we observed over woodland, an information‐dense surface environment (Zaleshina and Zaleshin 2018).

This conclusion regarding visual noise and speed does not, however, immediately clarify the changes seen when flying over urban space. It may have been the case that the areas we defined as urban did not constitute an excess of visual information. Although built‐up, the urban ground cover flown over by our birds was comprised of multiple fragmented areas, with marked ‘gaps’ of wooded or open ground (Figure 1). Given pigeons' apparent navigational preference for boundary habitats between urban and natural areas, and particularly for large linear landmarks such as roads or artificial treelines (Inglis et al. 1994; Holland 2003; Biro et al. 2004; Guilford et al. 2004; Lipp et al. 2004; Lau et al. 2006), it may even have been the case that our ‘sub‐urban’ built‐up areas offered intermediate levels of visual noise with large linear landmarks, optimal for route‐learning (Mann et al. 2014).

The reduction we observed in flap frequency over urban areas appears to have dissipated after approximately 10 flights (as per the positive 0.01 Hz interaction effect with flight iteration). Although not significant in comparison with open spaces, the increase of approximately 1.36 m/s flight speed over urban areas in comparison with wooded space similarly reduced by 0.09 m/s per flight. By the tenth flight, therefore, birds were flying slower and with higher flap frequency over urban spaces than they were at the start. Higher flap frequencies are believed to improve pigeons' perception of their surroundings during flight (Green et al. 1994; Ros and Biewener 2017; Taylor et al. 2019), so we do not believe this is evidence of improving route familiarity. Rather, we suspect it demonstrates the birds were becoming increasingly familiar with the short lengths of their flights, enabling them to adopt lower‐efficiency flight patterns (Rothe et al. 1987; Schwilch et al. 1996; Warrick and Dial 1998; Wiltschko et al. 2007; Williams and Biewener 2015). The reduced effect size over flight iteration we observed in flap frequency, flock spread, group size, and flight speed may be indicative of an increasing moderation of extremes over increased route familiarity. As birds develop stereotyped routes, flight route efficiency plateaus, alongside other heterogeneous flight parameters (Meade et al. 2005; Guilford and Biro 2014; Taylor et al. 2017).

### 4.4 Understanding Behaviour and Decision‐Making

In order to fully understand the reasoning behind any changes animals make apparently in response to their environment, a comprehensive awareness of all potentially involved factors (predator presence, visibility, weather conditions, noise levels, etc.) across all terrain being moved through is necessary. In practical terms, for the study of birds at least, this is an impossibility at present. An alternative that we advocate is the prioritisation of high‐detail recording of precise body movements and physiological changes in individual birds during flight over varied ground cover types (Wallraff 2001). Examples of fine‐scale body movements that might be enlightening include head position (Green et al. 1992; Kano et al. 2018, 2022; Taylor et al. 2019), neurophysiological condition (Vyssotski et al. 2006; Dennis et al. 2007; Mehlhorn and Rehkämper 2009; Hough 2022; Balanoff et al. 2024), or hormone levels (Schmidt‐Koenig 1963; George et al. 1989, 2010; Schwilch et al. 1996; Mehlhorn and Rehkämper 2009). Combining these with information on the habitats flown through might provide valuable, and quantifiable, insight into exactly why individuals are changing their behaviour in response to ground cover.

Additionally, we recommend that details of the sex of individuals assessed should be incorporated into future analyses, where possible. Sexual dimorphism is prevalent across pigeon breeds, with males being predominantly greater in body mass and size (Johnston 1990; Savaş and Erdem 2022). Data provided by Urquia‐Samele and Portugal (2025) report that male pigeons were consistently larger and heavier than females, and that resting metabolic rates were associated with body mass (irrespective of sex). It is unlikely, in our case, that both body mass and sex would be retained as variables in our models given the apparent overlap in explanatory power. However, homing pigeons can only be reliably sexed using DNA testing (Maciej et al. 2017), and we did not have this data on our birds. While we believe any sex‐related differences would likely be explained (or obscured) by body mass, it is possible that there were other undetected sex‐associated trends in the environmental responses of individuals; this is something observed in a range of species (Tropp and Markus 2001; Ball and Ketterson 2007; Ball 2016; Hodes 2018).

There may also have been differences in flight behaviour associated with the plumage colouration of the birds, as has been identified between different homing pigeon colour morphs in the past (Santos et al. 2015; Angelier et al. 2018; Frantz et al. 2025). The colour of wing feathers may impact the energetics of powered flight through, for example, changes to wing surface temperatures (Baker et al. 1997; Rogalla et al. 2022). Given the homogeneous mixture of colouration that our homing pigeons constituted, we do not believe our data are suitable for a focused analysis into the effect of plumage. We do, however, advocate the inclusion of plumage colouration as a consideration for future research when studying homing pigeons comprising a range of colour morphs.

### 4.5 Summary

To conclude, we found that homing pigeons altered both their flight patterns and flocking behaviour over changing ground cover. Flight speed was the variable most susceptible to changes in ground cover, with birds slowing down over woodland (as observed in previous research) and speeding up over urban areas. Birds appeared to adopt faster, but less defensive and energetically costly, gliding flight over built‐up urban areas, with a small but significant reduction in flap frequency. Small changes were also observed in flock spread and size over woodland, with fewer birds flying together cohesively, and further apart from each other. Whether the changes we observed had immediate energetic significance to our birds or not, they demonstrate that they did alter their behaviour in response to ground cover. Not only does this evidence of change have implications for longer flights over multiple ground cover types, such as that of migratory species, but may also belie other behavioural or neurological responses to a changing environment.

## Author Contributions

**Robin S. Mehlhausen‐Franks:** conceptualization (equal), data curation (lead), formal analysis (lead), funding acquisition (equal), investigation (lead), methodology (equal), project administration (supporting), visualization (lead), writing – original draft (lead), writing – review and editing (supporting). **Steven J. Portugal:** conceptualization (equal), funding acquisition (equal), investigation (supporting), methodology (equal), project administration (lead), resources (lead), supervision (lead), visualization (supporting), writing – review and editing (lead).

## Conflicts of Interest

The authors declare no conflicts of interest.

## Supporting information


**Appendix S1:** ece371902‐sup‐0001‐AppendixS1.zip.

## Data Availability

The raw data utilised for this project are openly available at https://doi.org/10.5061/dryad.4tmpg4fp6.
