# Supplementary material for: Changing Flight and Flocking Dynamics of Homing Pigeons (Columba livia d.) Over Heterogeneous Landscapes
Source: Ecol Evol. 2025 Aug 6;15(8):e71902. doi: 10.1002/ece3.71902 (PMC12326086; doi:10.1002/ece3.71902)
Supplement: Supplementary file 1 — Appendix S1: ece371902‐sup‐0001‐AppendixS1.zip. [file ECE3-15-e71902-s001.zip › ece371902-sup-0001-AppendixS1 Author Revisions/RSMF Figure & Appendix Legends Author Revised.docx]

1. Appendices
   1. Training of the Birds

Flocks L and R were fully comprised of birds that were newly purchased from private sellers on 25^th^ June 2022. With these birds having never been flown from their original lofts, an extensive training process was necessary to ensure the birds could reliably return to the home site when released and go into their respective lofts. The full training process comprised four stages: loft-training, increasing-distance releases, release-site releases and logger-training releases. The birds of flock N were experienced in homing flights and the use of their one-way loft doors, and so were not included in the first two phases.

After introduction to their respective lofts, flocks L and R were given 2 weeks of minimal human interaction to acclimatise to the lofts and to each other. This provided a period during which the flocks could establish social networks and hierarchies and reach a ‘resting’ social state. During the following 2 months, approximately 20 days of loft training were carried out to familiarise the birds with the surroundings of their lofts and the use of the one-way entrances. The birds were first shown the area carried around by hand, then placed in boxes abutted against the loft entrances, requiring them to learn the use of the doors in order to leave. After each bird had been through this process a minimum of seven times, staged ‘mini-releases’ were conducted from the roofs of the lofts. For each flock and on separate days, at first half of the flock was released from a carrier placed on top of the roof and then the full flock. This permitted the individual members of the flocks to freely explore the immediate surroundings and return inside their lofts, while mitigating the risk of flyaway losses had the full flocks been released in the first case.

Increasing-distance releases were conducted over the course of 1 month and comprised releases of lofts L and R from sites increasingly further from the home site: four releases from approximately 100 m, three releases from 400 m, one release from 1000 m, two releases from 3000 m, two from 6000 m, and one release from the experimental site at approximately 10,000 m. All of these sites were approximately in line with the flightpath from the final experimental release site to ensure familiarity with the appropriate landmarks and terrain (Figure S1). The subsequent release-site flights also occurred over approximately 1 month and were the first stage of training to include loft N. These comprised simple repeated releases of each flock from the release site, with L and R undergoing 11 flights and loft N 8 flights.

The final logger-training stage both acclimatised the birds to the mass and bulk of the biologgers they would be carrying during the experimental flights. After the birds were treated to carry loggers (detailed in Section 2b), they were released in their flocks directly from the home site and once from 1000 m without any other attachments. Then, 25 g weights were added to each bird's Velcro as dummy loggers (equivalent to the combined mass of the loggers), and they were released again once from the lofts and twice from 6000 m. Finally, the birds were equipped with their actual biologgers and flown again: once from their lofts, once from 4000 m, and once from 6000 m. The loggers were turned on during these flights, but their data were not retained beyond use for trial deployments.

- 1. Flight Altitude Range

Our GPS loggers did not record altitude to any degree of accuracy that was useful for statistical analysis but did provide data to calculate an approximate flight height range across all our flights. In order to identify minimum and maximum typical height ranges, we identified the lowest (non-negative) and highest values recorded in the raw data then calculated the medians of each. The raw data did include the time spent transporting the loggers by car, and so we discounted any values below 5 m. One bird (No. 29 from loft N) repeatedly spent extended periods of its flight resting on buildings at Windsor (approximately 51.482470, −0.607772), so this bird's data were also excluded from the calculation. Across all flights, the absolute minimum flight height recorded was 5.00 m, and the absolute maximum was 482.93 m. The median minimum value from across all data was 11.72 m, and the median maximum was 280.65 m.

- 1. Testing Models for Autocorrelation

Following the model selection process, we undertook visual assessment of residuals and autocorrelation function (ACF) plots and an additional modelling step to determine what impact autocorrelation had on our models. Linear mixed effects model (LMEM) equivalents of our GLMMs of best-fit were constructed with the *nlme* package version 3.1–163 (Pinheiro and Bates, [2000](#B178)) using the same fixed effects. Model construction with this package does not facilitate crossed random effects, only nested. For maximum comparability, we retained the bird as a random effect but removed loft due to its low effect size (variance < 0.01), although it was notably higher in the model for flight speed (variance = 0.14). The only exception to the removal of the loft random effect was group size, wherein loft was retained instead due to it having the larger effect (variance = 0.43). A correlation function of flight time (in its raw UTC form, not our added ascending counter of flight time) within each bird was included in the LMEMs, using the corCAR1 syntax for non-continuous timescales to facilitate missing data and inconsistent flight start and end times. All LMEMs detected the same small degree of autocorrelation (Phi = 0.2), and plotting of ACF did show varying degrees of autocorrelation, particularly in the readings of flight speed and group size (Figures [S2–S6](#F1)). We did not consider this sufficient evidence to reject the findings of our GLMM models; however, a comprehensive assessment of the LME outputs determined that there were only marginal changes to the model findings, with slight detrimental impact on model fit and no changes to any significance levels (Table S1).

Table S1. Output of a linear mixed effects (LME) remodelling of the generalised linear mixed effects model (GLMM) constructed through the model selection process, made using the *nlme* package.

| Dependent variable | Independent variable | Estimate | SE | *p* value |
| --- | --- | --- | --- | --- |
| Flap Frequency  (*N* = 6999,  AIC = 8430.15,  Phi = 0.2) | Urban Ground Cover | −0.10 | 0.03 | < 0.01 |
|  | Wooded Ground Cover | 0.03 | 0.03 | 0.36 |
|  | Flight Time | −0.14 | 0.01 | < 0.01 |
|  | Flight Iteration | < −0.01 | < 0.01 | 0.78 |
|  | Distance to Centroid | −0.02 | < 0.01 | < 0.01 |
|  | Group Size | 0.04 | 0.01 | < 0.01 |
|  | Flight Speed | −0.01 | < 0.01 | < 0.01 |
|  | Front-back Distance | −0.01 | < 0.01 | 0.02 |
|  | Front-Positioned | 0.01 | 0.01 | 0.37 |
|  | Urban Cover over Time | −0.01 | 0.02 | 0.56 |
|  | Wooded Cover over Time | 0.03 | 0.02 | 0.17 |
|  | Urban Cover over Iteration | 0.01 | < 0.01 | < 0.01 |
|  | Wooded Cover over Iteration | < 0.01 | < 0.01 | 0.58 |
| Absolute Flock Spread  (*N* = 6800,  AIC = 11641.92,  Phi = 0.2) | Urban Ground Cover | 0.06 | 0.04 | 0.12 |
|  | Wooded Ground Cover | 0.11 | 0.04 | < 0.01 |
|  | Flight Time | −0.02 | 0.01 | 0.26 |
|  | Flight Iteration | −0.01 | < 0.01 | < 0.01 |
|  | Distance to Centroid | 0.22 | < 0.01 | < 0.01 |
|  | Group Size | 0.20 | 0.01 | < 0.01 |
|  | Flight Speed | −0.01 | < 0.01 | 0.01 |
|  | Front-back Distance | 0.05 | 0.01 | < 0.01 |
|  | Front-Positioned | 0.06 | 0.02 | < 0.01 |
|  | Urban Cover over Time | 0.04 | 0.03 | 0.16 |
|  | Wooded Cover over Time | 0.01 | 0.03 | 0.72 |
|  | Urban Cover over Iteration | −0.01 | < 0.01 | 0.08 |
|  | Wooded Cover over Iteration | −0.01 | < 0.01 | 0.01 |
| Group Size  (*N* = 6615,  AIC = 18307.56,  Phi = 0.2) | Urban Ground Cover | −0.01 | 0.07 | 0.94 |
|  | Wooded Ground Cover | −0.17 | 0.06 | 0.01 |
|  | Flight Time | −0.62 | 0.02 | < 0.01 |
|  | Flight Iteration | −0.02 | < 0.01 | < 0.01 |
|  | Absolute Flock Spread | 0.08 | < 0.01 | < 0.01 |
|  | Distance to Centroid | −0.06 | 0.01 | < 0.01 |
|  | Flap Frequency | 0.16 | 0.03 | < 0.01 |
|  | Flight Speed | −0.01 | < 0.01 | < 0.01 |
|  | Urban Cover over Time | 0.03 | 0.04 | 0.53 |
|  | Wooded Cover over Time | 0.15 | 0.05 | < 0.01 |
|  | Urban Cover over Iteration | 0.01 | 0.01 | 0.15 |
|  | Wooded Cover over Iteration | 0.02 | 0.01 | 0.02 |
| Flight Speed  (*N* = 6696,  AIC = 35689.23,  Phi = 0.2) | Urban Ground Cover | 0.69 | 0.23 | < 0.01 |
|  | Wooded Ground Cover | −0.66 | 0.22 | < 0.01 |
|  | Flight Iteration | 0.02 | 0.01 | 0.20 |
|  | Absolute Flock Spread | −0.03 | 0.01 | < 0.01 |
|  | Group Size | −0.12 | 0.04 | < 0.01 |
|  | Flap Frequency | −0.35 | 0.09 | < 0.01 |
|  | Front-back Distance | 0.08 | 0.04 | 0.02 |
|  | Front-Positioned | −0.18 | 0.12 | 0.12 |
|  | Urban Cover over Iteration | −0.01 | 0.03 | 0.82 |
|  | Wooded Cover over Iteration | 0.08 | 0.03 | < 0.01 |
| Distance to Centroid  (*N* = 6386,  AIC = 9276.57,  Phi = 0.2) | Urban Ground Cover | 0.02 | 0.03 | 0.65 |
|  | Wooded Ground Cover | 0.02 | 0.03 | 0.55 |
|  | Flight Iteration | −0.01 | < 0.01 | < 0.01 |
|  | Flap Frequency | −0.06 | 0.01 | < 0.01 |
|  | Flight Speed | < −0.01 | < 0.01 | 0.06 |
|  | Front-back Distance | −0.06 | 0.01 | < 0.01 |
|  | Front-Positioned | 0.02 | 0.02 | 0.15 |
|  | Urban Cover over Iteration | −0.01 | < 0.01 | 0.15 |
|  | Wooded Cover over Iteration | < 0.01 | < 0.01 | 0.74 |

This model switched out Loft as a weak random effect for a continuous temporal correlation component (corCAR1), which was also found to be marginal (Phi = 0.2). Coefficient and significance estimates did not significantly differ from the original models in any of the fixed effects.

Figure S1. Map of sites involved in homing pigeon flight increasing-distance training. **1st – 4th:** Site of intermediate distance training sites from which birds were released in order, labelled with their displacement from the Home site. **Home:** Site of lofts where all birds were housed, to which birds homed. **Experimental**: The site from which birds were released during the experimental flights. Map created using *ggmap* version 4.0.0.

Figure S2. ACF (Autocorrelation Function, top) and residuals (bottom) of flap frequency values over flight time. Blue dotted lines indicate 95% confidence intervals for ACF, within which autocorrelation is considered insignificant. Red dotted lines indicate a y-intercept of flap frequency residual = 0, around which data is distributed. The ACF plot was created using the base-R ‘acf’ function, while the residuals plots were created using the *ggplot* R package.

Figure S3. ACF (Autocorrelation Function, top) and residuals (bottom) of absolute flock spread values over flight time. Blue dotted lines indicate 95% confidence intervals for ACF, within which autocorrelation is considered insignificant. Red dotted lines indicate a y-intercept of flock spread residual = 0, around which data is distributed. The ACF plot was created using the base-R ‘acf’ function, while the residuals plots were created using the *ggplot* R package.

Figure S4. ACF (Autocorrelation Function, top) and residuals (bottom) of group size values over flight time. Blue dotted lines indicate 95% confidence intervals for ACF, within which autocorrelation is considered insignificant. Red dotted lines indicate a y-intercept of group size residual = 0, around which data is distributed. The ACF plot was created using the base-R ‘acf’ function, while the residuals plots were created using the *ggplot* R package.

Figure S5. ACF (Autocorrelation Function, top) and residuals (bottom) of flight speed values over flight time. Blue dotted lines indicate 95% confidence intervals for ACF, within which autocorrelation is considered insignificant. Red dotted lines indicate a y-intercept of flight speed residual = 0, around which data is distributed. The ACF plot was created using the base-R ‘acf’ function, while the residuals plots were created using the *ggplot* R package.

Figure S6. ACF (Autocorrelation Function, top) and residuals (bottom) of distance to centroid values over flight time. Blue dotted lines indicate 95% confidence intervals for ACF, within which autocorrelation is considered insignificant. Red dotted lines indicate a y-intercept of distance to centroid residual = 0, around which data is distributed. The ACF plot was created using the base-R ‘acf’ function, while the residuals plots were created using the *ggplot* R package.

References

Pinheiro, J., and D. Bates. 2000. *Mixed-Effects Models in S and S-PLUS*. Springer Science & Business Media.
