# Supplementary figures and images for: Changing Flight and Flocking Dynamics of Homing Pigeons (Columba livia d.) Over Heterogeneous Landscapes
Source: Ecol Evol. 2025 Aug 6;15(8):e71902. doi: 10.1002/ece3.71902 (PMC12326086; doi:10.1002/ece3.71902)

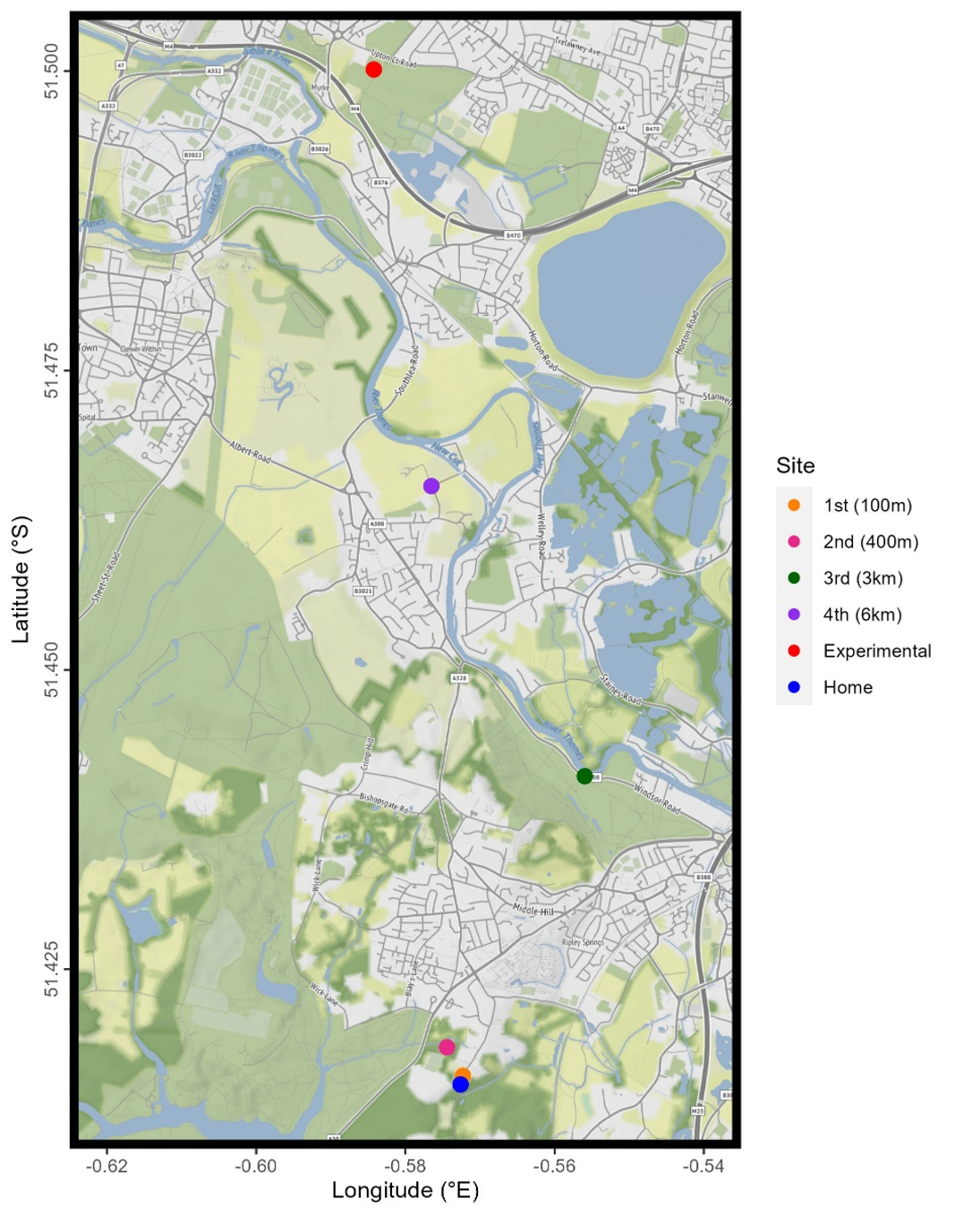

Supplement: Supplementary file 1 — Appendix S1: ece371902‐sup‐0001‐AppendixS1.zip. [file ECE3-15-e71902-s001.zip › ece371902-sup-0001-AppendixS1 Author Revisions/Appendix 1.tif]

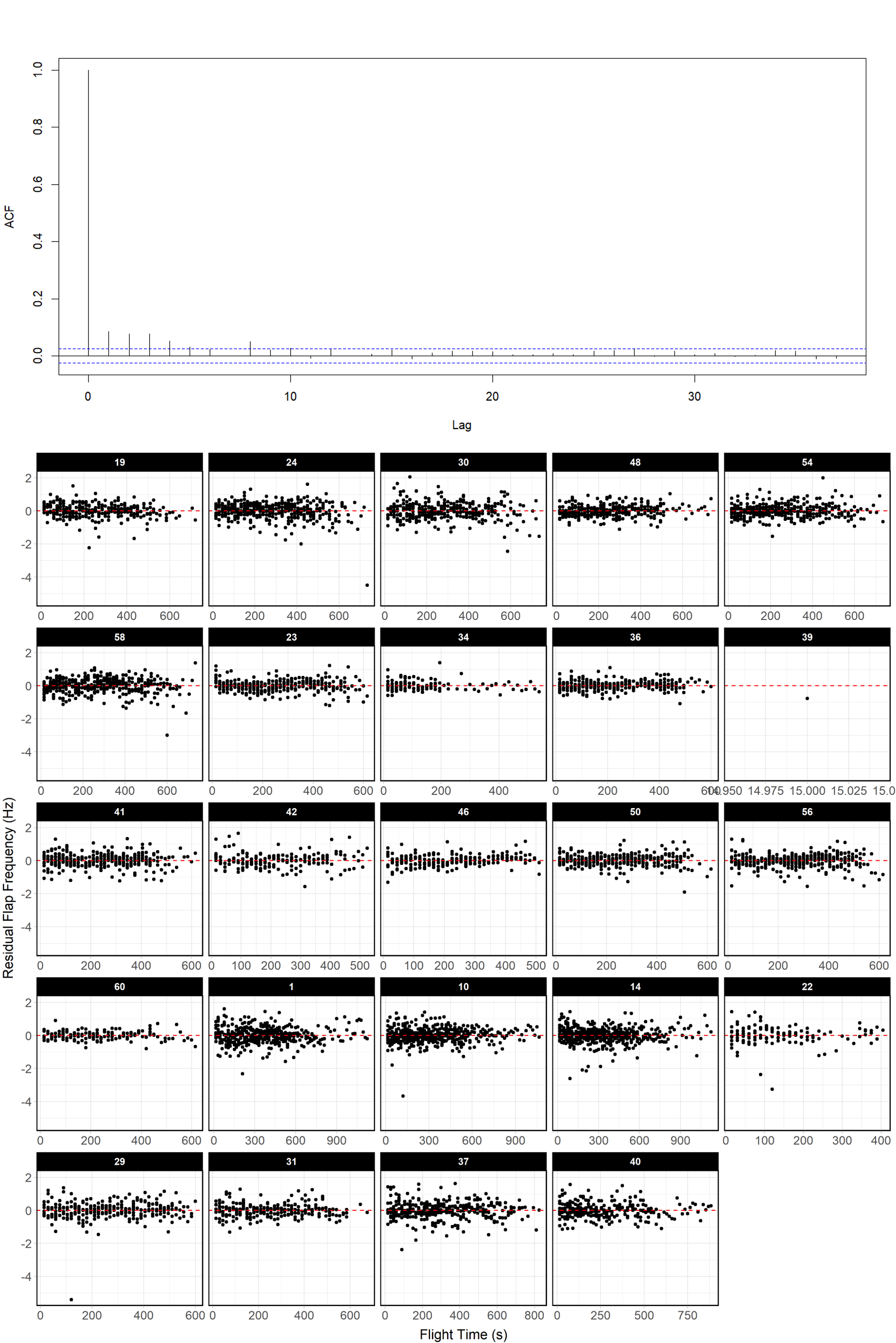

Supplement: Supplementary file 1 — Appendix S1: ece371902‐sup‐0001‐AppendixS1.zip. [file ECE3-15-e71902-s001.zip › ece371902-sup-0001-AppendixS1 Author Revisions/Appendix 2.tif]

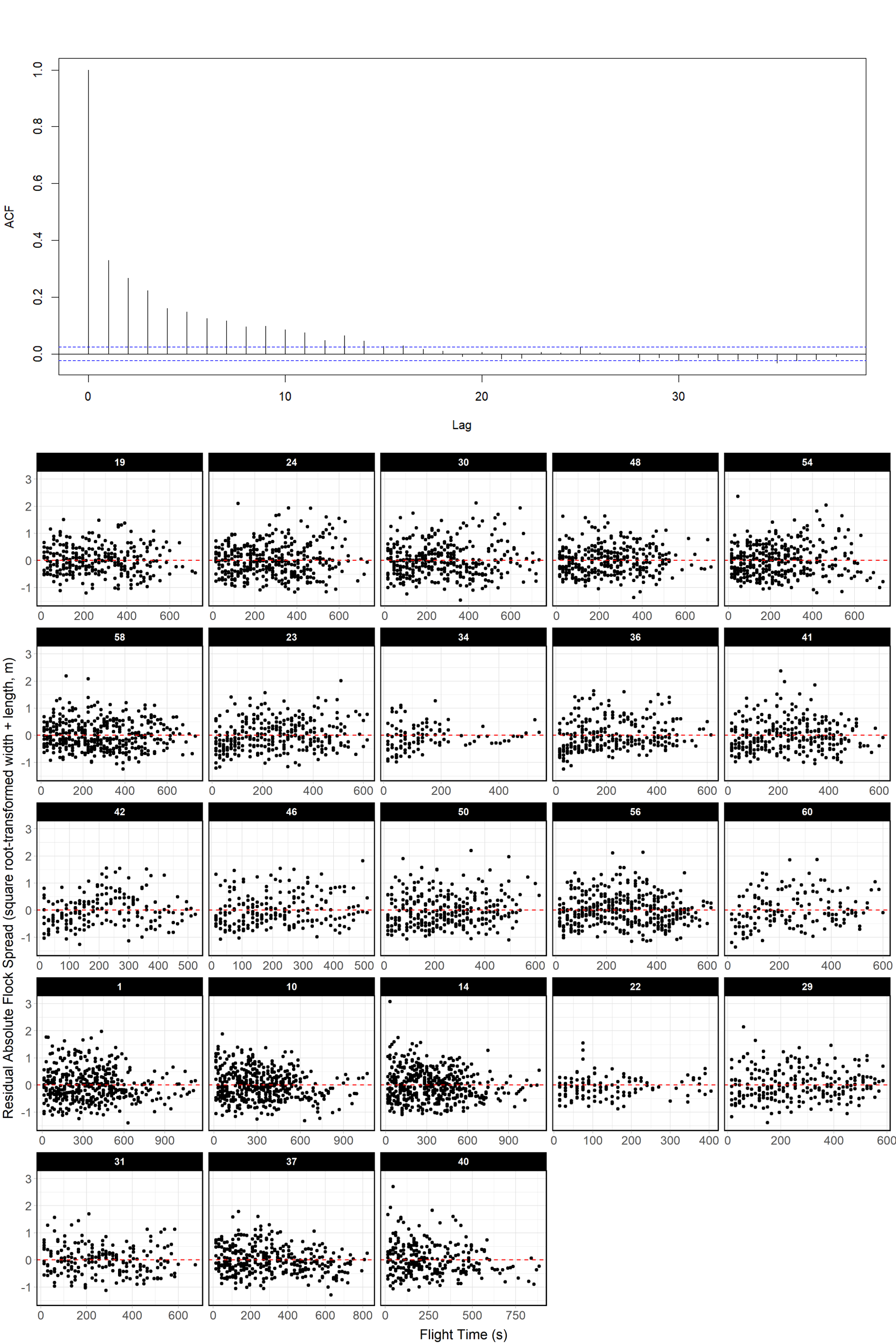

Supplement: Supplementary file 1 — Appendix S1: ece371902‐sup‐0001‐AppendixS1.zip. [file ECE3-15-e71902-s001.zip › ece371902-sup-0001-AppendixS1 Author Revisions/Appendix 3.tif]

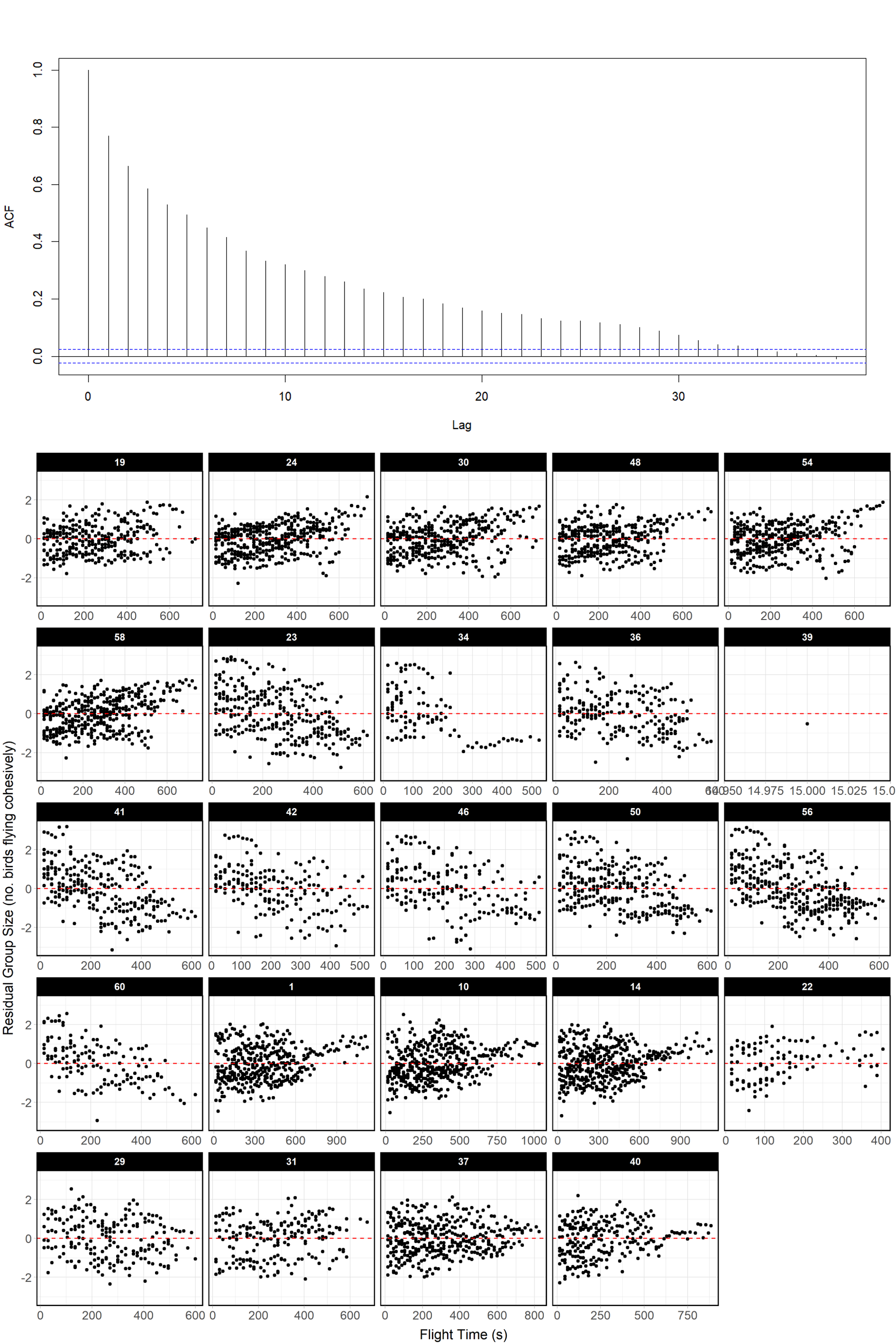

Supplement: Supplementary file 1 — Appendix S1: ece371902‐sup‐0001‐AppendixS1.zip. [file ECE3-15-e71902-s001.zip › ece371902-sup-0001-AppendixS1 Author Revisions/Appendix 4.tif]

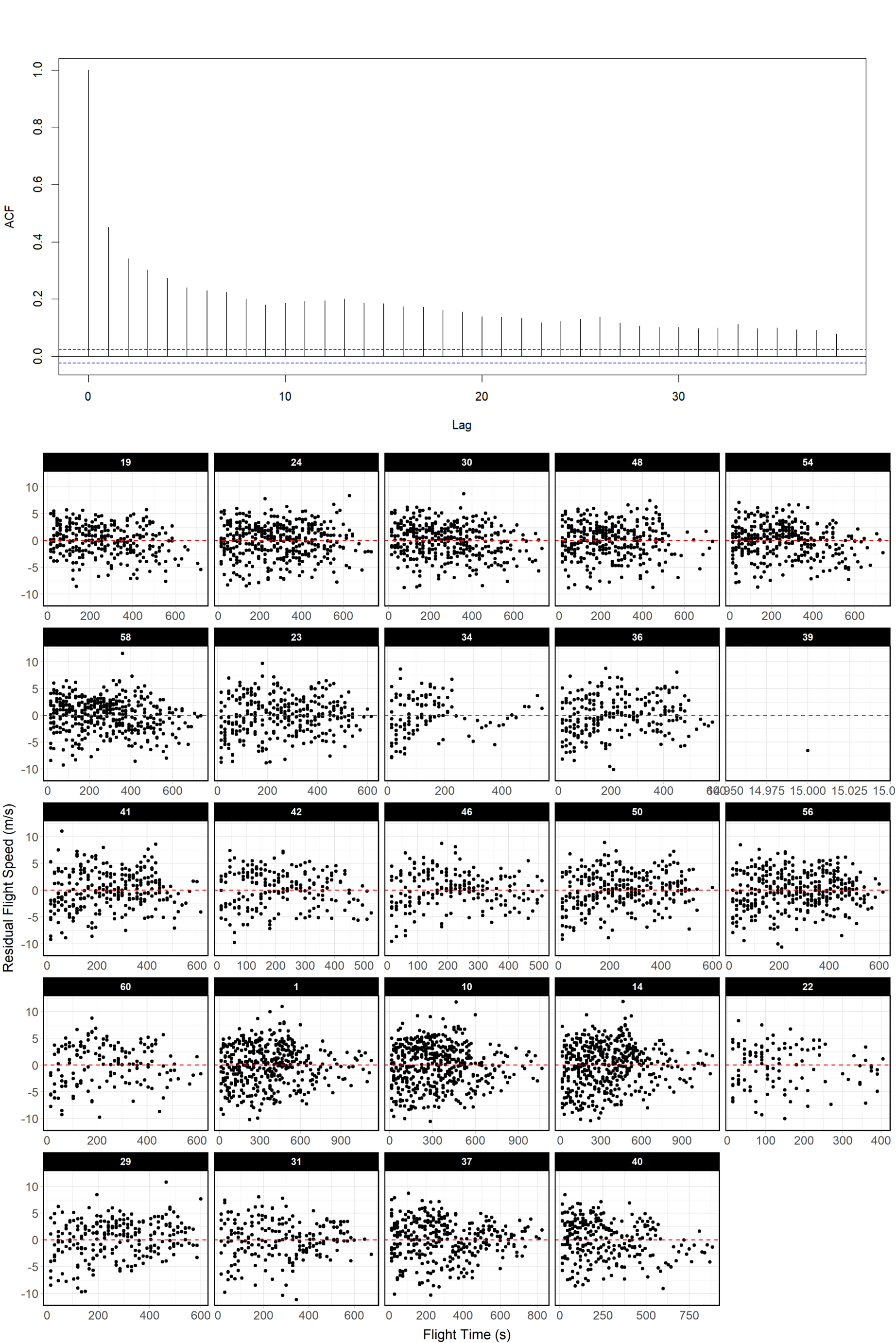

Supplement: Supplementary file 1 — Appendix S1: ece371902‐sup‐0001‐AppendixS1.zip. [file ECE3-15-e71902-s001.zip › ece371902-sup-0001-AppendixS1 Author Revisions/Appendix 5.tif]

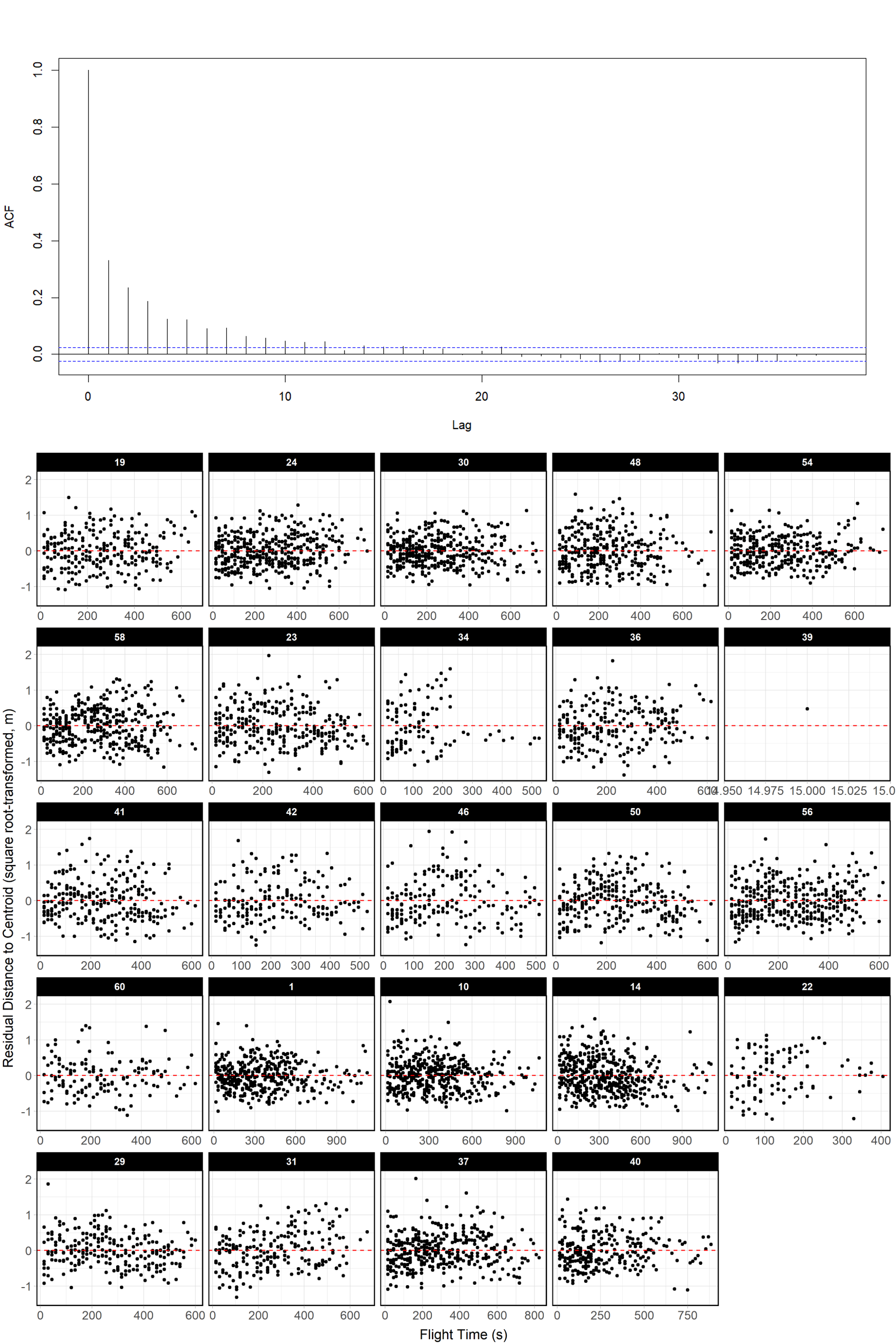

Supplement: Supplementary file 1 — Appendix S1: ece371902‐sup‐0001‐AppendixS1.zip. [file ECE3-15-e71902-s001.zip › ece371902-sup-0001-AppendixS1 Author Revisions/Appendix 6.tif]
